# Frontotemporal dementia characterization using neurite orientation dispersion and density imaging

**DOI:** 10.1093/braincomms/fcaf442

**Published:** 2025-11-11

**Authors:** Stefano Pisano, Silvia Basaia, Federica Agosta, Elisa Canu, Edoardo G Spinelli, Giordano Cecchetti, Alma Ghirelli, Elisa Sibilla, Giuseppe Magnani, Francesca Caso, Paola Caroppo, Sara Prioni, Cristina Villa, Lucio Tremolizzo, Ildebrando Appollonio, Federico Verde, Nicola Ticozzi, Vincenzo Silani, Massimo Filippi

**Affiliations:** Neuroimaging Research Unit, Division of Neuroscience, IRCCS San Raffaele Scientific Institute, 20132 Milan, Italy; Department of Medical Sciences and Public Health, University of Cagliari, 09042 Cagliari, Italy; Neuroimaging Research Unit, Division of Neuroscience, IRCCS San Raffaele Scientific Institute, 20132 Milan, Italy; Neuroimaging Research Unit, Division of Neuroscience, IRCCS San Raffaele Scientific Institute, 20132 Milan, Italy; Neurology Unit, IRCCS San Raffaele Scientific Institute, 20132 Milan, Italy; Vita-Salute San Raffaele University, 20132 Milan, Italy; Neuroimaging Research Unit, Division of Neuroscience, IRCCS San Raffaele Scientific Institute, 20132 Milan, Italy; Neuroimaging Research Unit, Division of Neuroscience, IRCCS San Raffaele Scientific Institute, 20132 Milan, Italy; Neurology Unit, IRCCS San Raffaele Scientific Institute, 20132 Milan, Italy; Vita-Salute San Raffaele University, 20132 Milan, Italy; Neuroimaging Research Unit, Division of Neuroscience, IRCCS San Raffaele Scientific Institute, 20132 Milan, Italy; Neurophysiology Service, IRCCS San Raffaele Scientific Institute, 20132 Milan, Italy; Neuroimaging Research Unit, Division of Neuroscience, IRCCS San Raffaele Scientific Institute, 20132 Milan, Italy; Neurology Unit, IRCCS San Raffaele Scientific Institute, 20132 Milan, Italy; Vita-Salute San Raffaele University, 20132 Milan, Italy; Neurophysiology Service, IRCCS San Raffaele Scientific Institute, 20132 Milan, Italy; Neuroimaging Research Unit, Division of Neuroscience, IRCCS San Raffaele Scientific Institute, 20132 Milan, Italy; Neurology Unit, IRCCS San Raffaele Scientific Institute, 20132 Milan, Italy; Neurology Unit, IRCCS San Raffaele Scientific Institute, 20132 Milan, Italy; Fondazione IRCCS Istituto Neurologico Carlo Besta, Unit of Neurology, 20133 Milan, Italy; Fondazione IRCCS Istituto Neurologico Carlo Besta, Unit of Neurology, 20133 Milan, Italy; Fondazione IRCCS Istituto Neurologico Carlo Besta, Unit of Neurology, 20133 Milan, Italy; Neurology Unit, “San Gerardo” Hospital and University of Milano-Bicocca, 20900 Monza, Italy; Neurology Unit, “San Gerardo” Hospital and University of Milano-Bicocca, 20900 Monza, Italy; Department of Neurology and Laboratory of Neuroscience, IRCCS Istituto Auxologico Italiano, 20149 Milan, Italy; Department of Neurology and Laboratory of Neuroscience, IRCCS Istituto Auxologico Italiano, 20149 Milan, Italy; “Dino Ferrari” Center, Department of Pathophysiology and Transplantation, Università degli Studi di Milano, 20122 Milan, Italy; Department of Neurology and Laboratory of Neuroscience, IRCCS Istituto Auxologico Italiano, 20149 Milan, Italy; “Dino Ferrari” Center, Department of Pathophysiology and Transplantation, Università degli Studi di Milano, 20122 Milan, Italy; Neuroimaging Research Unit, Division of Neuroscience, IRCCS San Raffaele Scientific Institute, 20132 Milan, Italy; Neurology Unit, IRCCS San Raffaele Scientific Institute, 20132 Milan, Italy; Vita-Salute San Raffaele University, 20132 Milan, Italy; Neurophysiology Service, IRCCS San Raffaele Scientific Institute, 20132 Milan, Italy; Neurorehabilitation Unit, Division of Neuroscience, IRCCS San Raffaele Scientific Institute, 20132 Milan, Italy

**Keywords:** NODDI, machine learning, FTLD spectrum

## Abstract

Microstructural alterations in brain tissue play a crucial role in the pathophysiology of frontotemporal dementia (FTD). This study assessed brain white matter (WM) and grey-matter (GM) microstructure in FTD variants using neurite orientation dispersion and density imaging (NODDI) diffusion MRI model and developed an exploratory machine-learning algorithm to classify FTD subtypes according to diffusion MRI metrics. Brain MRI including multi-shell diffusion sequences and neuropsychological assessment were obtained in controls and participants with FTD: 35 behavioural variant of FTD (bvFTD), 20 semantic-variant primary progressive aphasia (svPPA), 14 nonfluent-variant primary progressive aphasia (nfvPPA), 9 semantic-bvFTD (sbvFTD). Fractional anisotropy (FA), mean diffusivity (MD), intracellular-volume fraction (ICVF), and orientation-dispersion index (ODI) were analysed using tract-based and GM-based spatial statistic at the voxel-wise level, with nonparametric and permutation-based methods. Support vector machine (SVM) models were trained on different combinations of diffusion MRI and neuropsychological features to classify FTD subtypes. FA and MD showed widespread WM alterations in all variants. ICVF showed reductions in both WM and GM (bilateral frontotemporal for bvFTD, left temporal-frontal for svPPA and nfvPPA and right temporal for sbvFTD). GM ODI reduction exhibited a similar but more diffuse pattern compared with ICVF. WM ODI alterations were also observed, with specific WM alterations in the corpus callosum and long-range WM tracts when comparing FTD syndromes. SVM algorithm, trained on mean FA, ICVF and ODI values from different brain lobes and neuropsychological scores, achieved 98.6% accuracy in classifying different clinical syndromes, outperforming standard diffusion tensor (DT) imaging-based models. NODDI capture subtle microstructural alterations in brain GM and WM, demonstrating advantages over standard DT imaging in capturing disease-relevant alterations. By integrating NODDI with cognitive data, machine-learning models can learn complex patterns and relationships facilitating the differentiation of FTD subtypes.

## Teaser

Pisano *et al*. report that neurite orientation dispersion and density imaging diffusion magnetic resonance imaging reveals distinct spatial microstructural patterns in white and grey matter of frontotemporal dementia variants, and developed a multimodal machine-learning model that classified subtypes with 97.3% accuracy, outperforming standard diffusion tensor imaging.

## Graphical Abstract

**Graphical Abstract:**
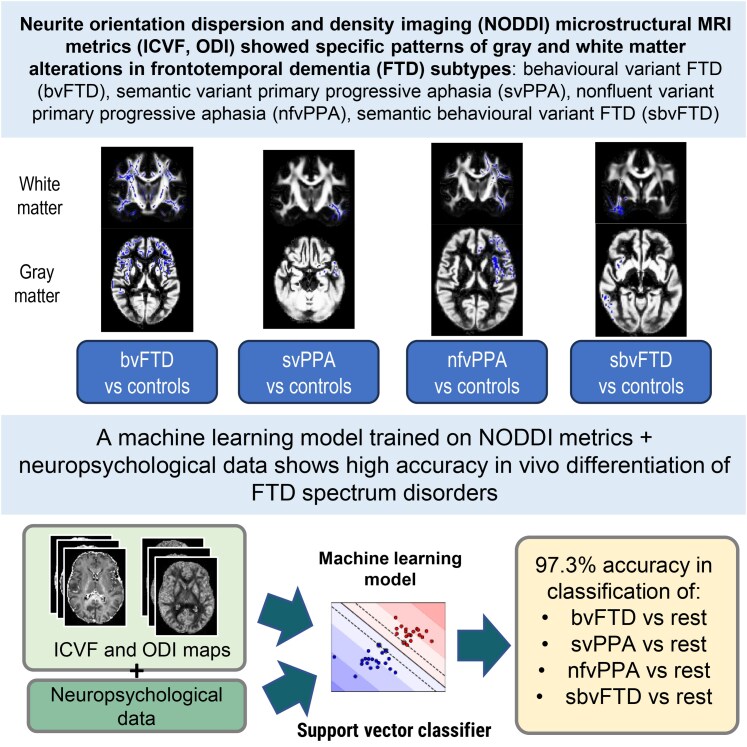

## Introduction

Frontotemporal dementia (FTD) is a group of heterogeneous neurodegenerative disorders characterized by progressive cognitive decline and prominent behavioural, executive and/or language impairment.^1^ FTD encompasses a spectrum of syndromes that includes behavioural variant of FTD (bvFTD),^2^ nonfluent/agrammatic variant of primary progressive aphasia (nfvPPA) and semantic variant of primary progressive aphasia (svPPA).^3^ More recently, a syndrome with predominant degeneration in right anterior temporal lobe has been identified and described as semantic-behavioural variant FTD (sbvFTD).^4^

In recent years, diffusion MRI techniques, such as diffusion tensor imaging (DTI), have emerged as promising noninvasive tools for assessing microstructural alterations associated with neurodegeneration.^5,6^ Several studies explored DTI alterations in FTD, demonstrating white matter (WM) tract damage with widespread changes in bvFTD, more focal damage in the left temporal lobe in svPPA, and in the left temporal and frontal regions in nfvPPA patients.^7-9^ To date, no cross-sectional studies have directly characterized DTI-based microstructural alterations in sbvFTD. Only a recent study has applied network diffusion models based on healthy controls’ (HC) structural connectome [fractional anisotropy (FA), intracellular-volume fraction (ICVF) and orientation-dispersion index (ODI)] to predict the spread of atrophy across different FTD subtypes, including sbvFTD.^10^

Although DTI has provided valuable information about microstructural alterations in neurodegenerative disorders, its single compartment model has some limitations, such as biases introduced by partial volume effect,^11^ crossing fibres^12^ and lack of biological specificity of DTI measures.^13^ To overcome some of these issues, a multi-shell diffusion MRI technique called neurite orientation dispersion and density imaging (NODDI) has been introduced.^14^ NODDI is a biophysical model assuming that each voxel’s microstructural characteristic can be described by a combination of three compartments with different water diffusion properties: intra-neurite, extra-neurite, and cerebrospinal fluid. By modelling the contribution of such compartments to each voxel, NODDI can estimate different measures, such as ICVF and ODI. Such measures are biologically interpretable, having shown correlation with histological-derived metrics.^15,16^ Another big advantage of NODDI model over standard DTI is its ability to better describe grey-matter (GM) complexity by capturing the heterogeneous microstructural properties within GM regions.^17^ Up to date, NODDI has been useful to evaluate *in vivo* WM and GM damage in different neurodegenerative disease (such as Alzheimer’s disease, Parkinson’s disease and amyotrophic lateral sclerosis), providing information concerning different aspects of cortical microstructure.^18-20^ Only one preliminary study investigated the benefits of NODDI in the evaluation of WM damage in FTD syndromes, showing widespread WM involvement in sporadic forms.^21^

With such a framework, the aim of this study was to evaluate the patterns of NODDI-derived measures alterations in both WM and GM across the FTD spectrum. We aimed to identify distinctive patterns of microstructural changes associated with each FTD clinical subtype, exploring the utility of NODDI as a noninvasive biomarker for the detection and differential diagnosis of FTD, and comparing its performance with DTI. Additionally, we investigated the implementation of machine-learning algorithms that integrate NODDI-derived metrics with clinical neuropsychological data, to develop a robust diagnostic tool capable of differentiating FTD syndromes.

## Materials and methods

This is a prospective study which was approved by the local ethical standard committee on human experimentation and all patients provided written informed consent prior to study inclusion.

### Participants

One hundred sixty-five patients with a suspected FTD-spectrum clinical syndrome were referred to the Neurology Unit of San Raffaele Hospital (Milan, Italy) from 2017 to date, to perform a comprehensive evaluation,^22,23^ including a thorough neurological and neuropsychological assessment, as well as a 3.0 T brain MRI incorporating diffusion sequences. This multidisciplinary evaluation allowed to identify 130 patients with bvFTD, svPPA, nfvPPA and sbvFTD who were subsequently evaluated for inclusion in the present cross-sectional study. Inclusion criteria were: diagnosis of bvFTD, svPPA, nfvPPA and sbvFTD according to international diagnostic criteria.^2-4^ Exclusion criteria were: overlapped amyotrophic lateral sclerosis; significant medical illnesses or substance abuse that could interfere with cognitive functioning; any (other) major systemic, psychiatric or neurological illnesses; lacunae and extensive cerebrovascular disorders at MRI; significant artefacts on MRI or missing T_1_-weighted, T_2_-weighted or diffusion MRI sequences. Twenty-seven patients were excluded due to the aforementioned criteria. In addition, in order to mitigate sources of sample heterogeneity, after screening for known pathogenic mutations (see below for details), 25 patients with known pathogenic mutations (i.e. 16 GRN, 5 C9orf72, 2 MAPT, 1 C9orf72 + MAPT and 1 TREM2) were excluded, due to different spatial and temporal patterns of atrophy and degeneration.^24,25^ The final study cohort included 78 participants with sporadic FTD: 35 bvFTD, 20 svPPA, 14 nfvPPA and 9 sbvFTD (age range: 48–81 years). A subsample of 39 patients (20 bvFTD, 7 svPPA, 7 nfvPPA and 5 sbvFTD) also underwent lumbar puncture for cerebrospinal fluid biomarker dosage (Aβ42, tTau and pTau); no patients showed an Alzheimer's disease-like biomarker profile based on the pTau/Aβ42 ratio.

Forty-eight HC, comparable for age and sex with patients, were recruited among spouses of patients and by word of mouth. Inclusion criteria for the control group were: normal neurological assessment, Mini-Mental State Examination (MMSE)^26^ score ≥26, no family history of neurodegenerative diseases. Exclusion criteria for HC were: any significant systemic, psychiatric and neurological diseases, and focal or diffuse brain damage, including ischaemic lacunae and extensive cerebrovascular lesions visible on MRI scans.

All patients and controls were previously included in two studies.^22,23^ These prior articles dealt with functional connectivity patterns in FTD, whereas in the present study we report on structural connectivity assessed with NODDI.

### Clinical and cognitive assessment

Clinical evaluation was performed by experienced neurologists, recording disease duration at presentation. Global disease severity was assessed using the Clinical Dementia Rating (CDR) and the CDR-sum of boxes (CDR-SB).^27^ Participants also underwent a comprehensive neuropsychological assessment. Details of cognitive and behavioural assessment are fully reported in the Supplementary material.^22,23,28^

### Genetic testing

The presence of pathological C9orf72 expansions and/or known pathogenic variants in the GRN, MAPT, FUS, TARDBP, TBK1, TREM2, OPTN and VCP genes was assessed from blood samples using optimized protocols.^24^

### MRI acquisition

All participants underwent a brain MRI scan on a 3.0 Tesla Philips Ingenia CX scanner (Philips Medical Systems) with standardized procedures for subjects’ positioning and repositioning. The protocol included T_2_-weighted, 3D fluid-attenuated inversion recovery (FLAIR), 3D high resolution T_1_-weighted sequence and axial pulsed-gradient spin echo single-shot diffusion-weighted (DW) echo-planar imaging sequence. Details about MRI acquisition protocols are provided in Supplementary material.

### MRI analysis

**Figure 1:**
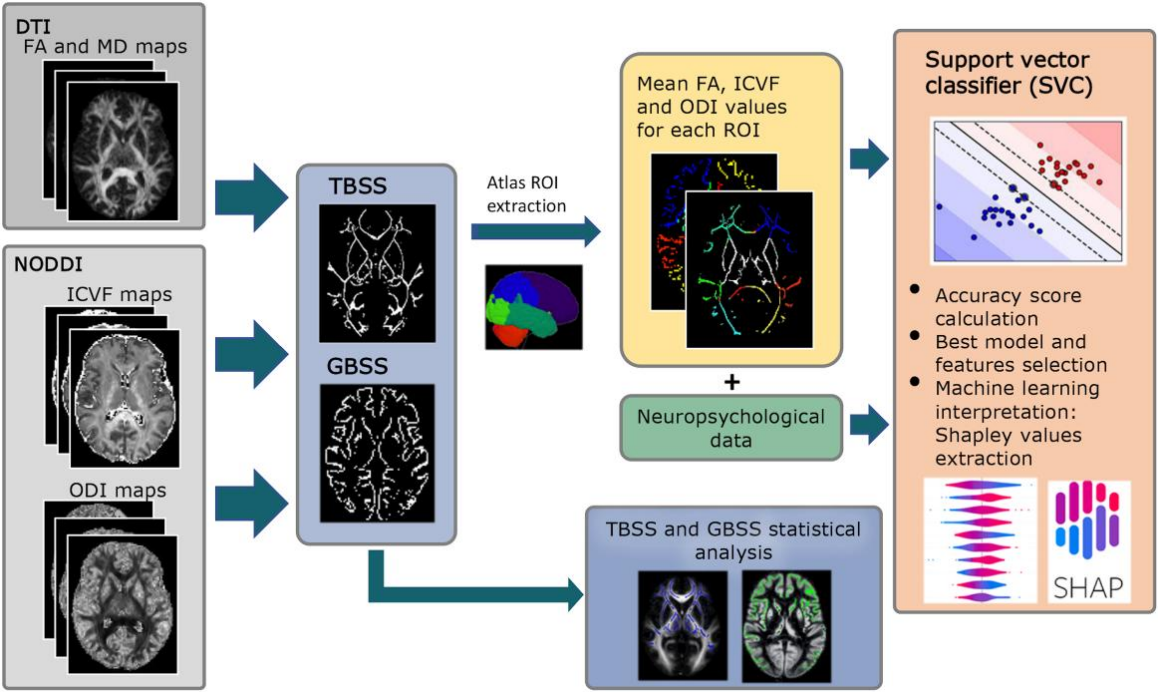
**Schematic diagram representing the MRI analysis pipeline.** Individual maps of FA, MD, ICVF and ODI (on the left side in the picture) were measured on NODDI sequence. TBSS and GBSS preprocessing was applied on individual WM and GM maps resulting in 4D-WM and –GM images, respectively. For each subject, mean values of skeletonized FA (WM only), MD (WM only), ICVF and ODI from WM/GM maps were obtained for each different region of interest of the USC Lobes brain atlas and considered as features for the subsequent machine-learning analysis. A classifier model (on the right side in the picture), trained on MRI and neuropsychological features, was constructed to identify hyperplanes which maximize the distance between subjects belonging to different clinical FTD syndromes. FA, fractional anisotropy; MD, mean diffusivity; GBSS, grey-matter-based spatial statistics; ICVF, intracellular fractional volume; L, left; ODI, orientation-dispersion index; ROI, region of interest; TBSS, tract-based spatial statistics.

A schematic diagram representing the MRI analysis pipeline is reported in Fig. 1.

#### Voxel-based morphometry

Voxel-based morphometry (VBM) was used to assess local patterns of GM atrophy in the different clinical groups. The analysis was performed using Statistical Parametric Mapping (SPM12) (http://www.fil.ion.ucl.ac.uk/spm/), using Diffeomorphic Anatomical Registration Exponentiated Lie Algebra (DARTEL) as the registration method (see Supplementary material for details).^29^

#### Diffusion-weighted MRI analyses

Preprocessing of DW imaging included correction for off-resonance and eddy current-induced distortions, as well as for movement, outlier detection and replacement using the Eddy tool within the FSL library. The process is described in the Supplementary material.^30^ The diffusion tensor (DT) was estimated by linear regression using a multi-shell approach (three shells, with *b* = 700, 1000 and 2855 s/mm^2^), using the dtifit tool implemented in FSL. Subsequently, FA maps were derived. For the NODDI model, ICVF and ODI maps were computed using the NODDI Toolbox v0.9 executed within a MATLAB R2023 environment with default settings (http://www.nitrc.org/projects/noddi_toolbox). Isotropic volume fraction (IsoVF) was not included in this study due to technical and biological considerations. IsoVF estimates are particularly susceptible to partial volume effects with CSF, which can be challenging to rigorously address in regions with pronounced atrophy, as seen in advanced FTD.^15,16^

#### Tract-based spatial statistics analysis

Voxel-wise DT MRI analysis was performed using tract-based spatial statistics (TBSS) tool implemented in FSL (version 5.0.9) (http://www.fmrib.ox.ac.uk/fsl/fdt/index.html) to obtain FA, mean diffusivity (MD), ICVF and ODI diffusion skeletonized WM maps. Details of the TBSS pipeline are described in Supplementary material.^31^ Four different 4D WM images (with all the subjects) were created including FA, MD, ICVF and ODI measures. To perform the machine-learning analysis, the 4D maps were then coregistered to USCLobes brain atlas (http://brainsuite.org/usclobes-description).^32,33^ For each subject, mean values of skeletonized FA, MD, ICVF and ODI from WM maps were obtained for each region of interest (ROI) of the USCLobes brain atlas and considered as features for the correlation and machine-learning analyses. Brainstem and cerebellum ROIs were discarded from subsequent analysis.

#### Grey-matter-based spatial statistics analysis

Grey-matter-based spatial statistics (GBSS) is a technique to assess voxel-wise differences in GM microstructure.^34,35^ Details about the procedure are described in Supplemental material. Six subjects were excluded from GBSS analysis due to coregistration errors (one control, two bvFTD, two svPPA and one nfvPPA). The process returns skeletonized maps of ICVF and ODI on the GM. For each subject, mean values of skeletonized ICVF and ODI from GM maps were obtained for ROI of the USCLobes brain atlas and considered as features for the correlation and machine-learning analyses. Brainstem and cerebellum ROIs were discarded from subsequent analysis. DTI-derived metrics such as FA and MD were not included in this analysis due to the sensitivity of the DTI model to partial volume effects, which can compromise reliability in the context of GM analysis.

### Statistical analysis

#### Demographic, clinical, and cognitive data

Demographic, clinical and cognitive/behavioural data were compared between FTD groups and HC. ANOVA with *post hoc* test was used for continuous variables (correcting *P*-values for multiple comparisons using the Bonferroni method) and *χ*^2^ test for categorical variables. Two-sided *P*-value < 0.05 was considered for statistical significance. Statistical analysis was performed using the R software.

#### VBM, TBSS and GBSS voxel-wise analysis

Voxel-wise differences in regional atrophy between groups were evaluated using an analysis of co-variance (ANCOVA) model with age, sex, education and total intracranial volume as covariates (parametric test performed with SPM12). Results were assessed at *P* < 0.05, correcting for multiple comparisons with family-wise error. Voxel-wise statistics on TBSS and GBSS skeletonized maps, comparing the different groups, was performed using a nonparametric, permutation-based, inference tool (‘randomise’, part of FSL), using age, sex and education as covariates with 5000 permutations. The results were corrected for multiple comparisons with the threshold-free cluster enhancement, and displayed with a *P* < 0.05.

#### Correlation analysis

Partial correlations were performed to explore the relationship between mean ICVF, ODI, MD and FA values from different ROIs and global scores of disease severity (CDR-SB) and cognitive function (MMSE) in the FTD groups. These analyses accounted for potential confounders including age, sex and education level of each participant. Additionally, to address the issue of multiple comparisons, a Bonferroni correction was applied (*P* < 0.05).

#### Machine learning

Multiple classifier models were trained on different combinations of 76 MRI features [mean FA (only WM), MD (only WM), ICVF (WM + GM) and ODI (WM + GM) values of the USCLobes ROIs] and 9 selected neuropsychological tests (see Supplementary material for details). Different models were trained on different combinations of the scaled features, optimizing on accuracy score. For each model, a cross-validation procedure was performed by using the leave-one-out method. This procedure estimates the performance of the machine-learning model on data not used for the training, iteratively creating a model for each subject in the dataset, leaving the subject out of the training data and using it to test the algorithm, thus reducing the risk of estimating falsely elevated accuracies and reducing the risk of overfitting the model. The performances of the different models were evaluated using the following measures: (i) accuracy score (i.e. the fraction of correct predictions out of the totals); (ii) precision (known as positive predictive value); (iii) recall (i.e. sensitivity) and (iv) *F*-score (i.e. a combination of precision and recall). To establish a baseline for performance comparison, we implemented the use of two dummy classifiers. They made predictions choosing a random class for each patient and predicting the most frequent class (bvFTD), respectively. By comparing our model’s performance with these classifiers, we can ascertain that the model is effectively capturing meaningful patterns within our data. This comparison is particularly valuable when dealing with imbalanced class distribution, as is the case in our study. If a machine-learning model outperforms a dummy classifier’s performance, it indicates that the model has learned valuable information from the features, and it can make informed prediction.

Finally, in order to improve the classification accuracy, a backward sequential feature selection algorithm was applied to support vector machine (SVM) models, systematically evaluating the relevance and contribution of each feature to the model’s performance and excluding the ones with a negative impact. This process enables to identify the most discriminative and influential features, resulting in an improved accuracy compared with the initial model.

To facilitate interpretability of the best performing model, the SHapley Additive exPlanations (SHAP) method was applied.^36^ The most important features used by the model for the classification were calculated. Transitioning to a single subject-perspective, this process allowed assessing the importance of each feature in subject-specific classification and leading to a better understanding of the role of individual features and their influence on syndrome classification. Considering the small size of the participants' cohort, linear machine-learning algorithms like SVM are advantageous due to their simplicity, which helps mitigate the risk of overfitting compared with methods that are more complex. Nevertheless, in order to prove this hypothesis, we applied other machine-learning techniques (e.g. random forest, Naive Bayes, XGBoost and logistic regression) to our data.

## Results

### Demographic, clinical and cognitive data

One hundred sixty-five patients were initially recruited. After exclusions of subjects according to exclusion criteria, 78 participants with sporadic FTD (35 bvFTD, 20 svPPA, 14 nfvPPA and 9 sbvFTD; mean age range at MRI, 48–81 years) and 48 HC (mean age at MRI, 62 years) were included. No evidence of a difference between participants with FTD and controls was found for age (*P* range = 0.28–1.00), sex (*P* range = 0.08–0.98) and education (*P* range = 0.26–1.00) (Table 1). However, participants with bvFTD were older than controls (*P* = 0.01) and participants with sbvFTD presented a higher number of males than nfvPPA (*P* = 0.04). Moreover, participants with bvFTD showed higher CDR (*P* = 0.02) and CDR-SB (*P* = 0.01) relative to nfvPPA cases. Disease duration was longer in participants with sbvFTD relative to nfvPPA (*P* = 0.02) (Table 1). Results of the neuropsychological assessment are reported in Supplementary Table 1.

**Table 1:** Demographic and main clinical characteristics of included subjects

	HC	bvFTD	svPPA	nfvPPA	sbvFTD
*N*	48	35	20	14	9
Age at MRI (years)^a^	61.92 ± 8.56 (40.14–76.73)	68.36 ± 7.12* (55.68–79.77)	66.95 ± 8.79 (48.46–81.63)	67.45 ± 9.27 (51.76–79.04)	62.90 ± 11.26 (48.36–77.15)
Sex					
Males	32	19	10	5	8^§^
Females	16	16	10	9	1
Education (years)^a^	12.23 ± 4.16 (5.00–23.00)	10.03 ± 3.53 (5.00–18.00)	13.05 ± 4.30 (5.00–18.00)	11.67 ± 5.74 (4.00–22.00)	9.33 ± 2.65 (5.00–13.00)
Disease duration (years)^a^		3.44 ± 2.13 (1.06–8.64)	3.61 ± 2.35 (0.94–10.55)	1.42 ± 0.92 (0.12–2.72)	4.98 ± 2.99^§^ (1.46–8.23)
CDR^a^		1.17 ± 0.89^§^ (0.00–3.00)	0.67 ± 0.58 (0.00–2.00)	0.42 ± 0.60 (0.00–2.00)	0.94 ± 0.73 (0.00–2.00)
CDR-SB^a^		6.54 ± 4.82^§^ (1.00–18.00)	3.35 ± 3.44 (0.5–10.5)	2.17 ± 2.35 (0.00–8.5)	6.19 ± 4.92 (1–14.00)

Categorical variables are presented as the number of participants. Groups were compared using Pearson’s *χ*^2^. The threshold of statistical significance was set at *P* < 0.05. bvFTD, behavioral variant frontotemporal dementia; CDR, Clinical Dementia Rating; CDR-SB, Clinical Dementia Rating sum of boxes; HC, healthy controls; nfvPPA, nonfluent/agrammatic variant primary progressive aphasia; sbvFTD, semantic behavioral variant frontotemporal dementia; svPPA, semantic-variant primary progressive aphasia. ^a^Continuous data are reported as mean ± standard deviation (min—max) and groups were compared using ANOVA models followed by *post hoc*. *Statistically significant difference with HC. ^§^Statistically significant difference with nfvPPA.

### Voxel-based morphometry

Compared with controls, all disease groups exhibited patterns of GM atrophy in expected areas. Results are shown in Supplementary Fig. 1 and described in Supplementary material.

### Tract-based spatial statistics

**Figure 2:**
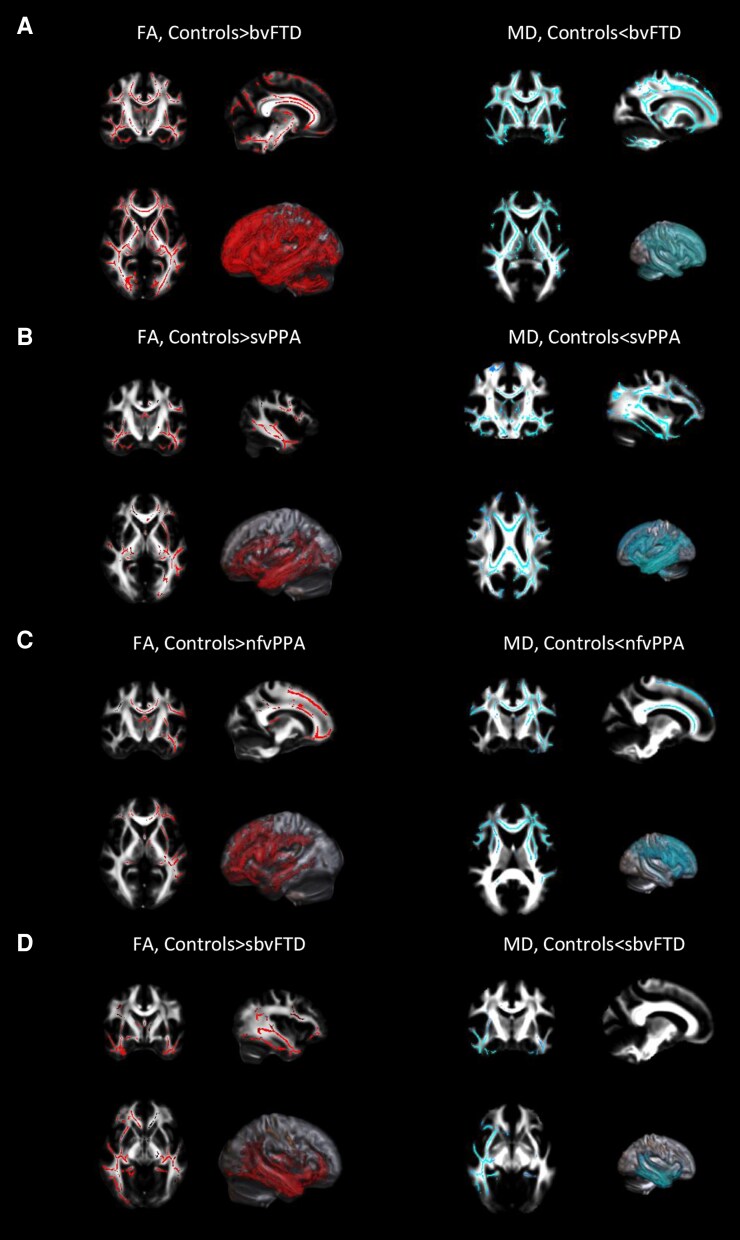
**TBSS of DTI-derived microstructural metrics in FTD variants relative to controls.** TBSS results in patients with bvFTD (**A**), in patients with svPPA (**B**), in patients with nfvPPA (**C**), and in sbvFTD patients (**D**) versus healthy controls. Results are overlaid on the axial, coronal and sagittal sections of the Montreal Neurological Institute standard brain in radiological convention (right is left), and displayed at *P* < 0.05 corrected for multiple comparisons, adjusting for age, sex and education. L, left; R, right.

**Figure 3:**
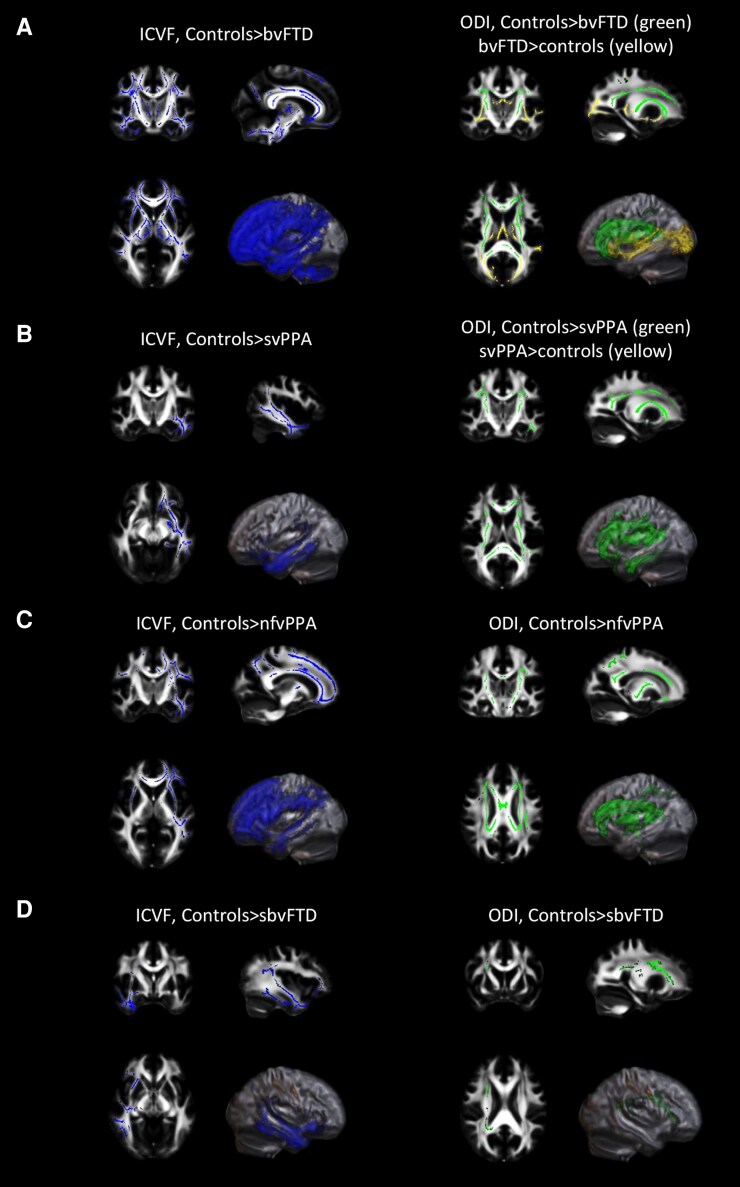
**TBSS of NODDI-derived microstructural metrics in FTD variants relative to controls.** TBSS results in patients with bvFTD (**A**), in patients with svPPA (**B**), in patients with nfvPPA (**C**), and in sbvFTD patients (**D**) versus healthy controls. Decreased ICVF is shown in blue, decreased ODI is shown in green and increased ODI is shown in yellow. Results are overlaid on the axial, coronal and sagittal sections of the Montreal Neurological Institute standard brain in radiological convention (right is left), and displayed at *P* < 0.05 corrected for multiple comparisons, adjusting for age, sex and education. L, left; R, right.

Significant results (*P* < 0.05) are shown in Figs 2 and 3 (all patient groups relative to controls) and Supplementary Tables 2–5 and described in details in Supplementary material. Mean values of FA, MD, ICVF and ODI, as obtained from WM ROIs, are reported in Supplementary Table 6.

Briefly, each group showed specific pattern of brain WM changes. bvFTD showed widespread bilateral reductions in FA, ICVF and ODI and increased MD relative to controls, involving mainly bilateral frontotemporal tracts when compared with other patient groups. svPPA and nfvPPA had left-sided predominant FA and ICVF reductions and increased MD in frontotemporal regions compared with controls. sbvFTD showed FA, MD and ICVF alterations primarily in right temporal areas relative to controls. ODI reductions were also observed in different brain regions for each group relative to controls, with specific WM alterations in the corpus callosum and long-range WM tracts when comparing FTD syndromes.

### Grey-matter-based spatial statistics

**Figure 4:**
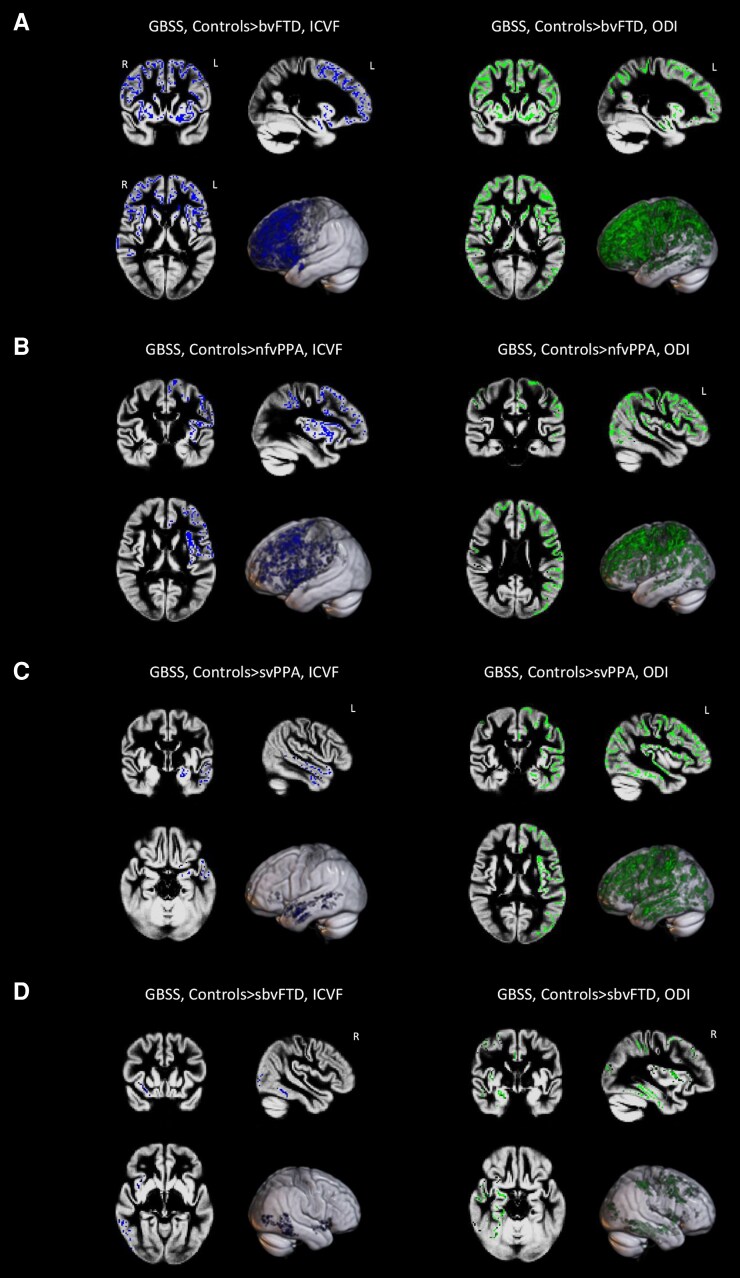
**GBSS of NODDI-derived microstructural metrics in FTD variants relative to controls.** GBSS results in patients with bvFTD (**A**), in patients with svPPA (**B**), in patients with nfvPPA (**C**) and in sbvFTD patients (**D**) versus healthy controls. Results are overlaid on the axial, coronal and sagittal sections of the Montreal Neurological Institute standard brain in radiological convention (right is left), and displayed at *P* < 0.05 corrected for multiple comparisons, adjusting for age, sex and education. L, left; R, right.

Significant results (*P* < 0.05) are shown in Fig. 4 (all patients groups relative to controls) and Supplementary Tables 7 and 8, and described in details in Supplementary material. Mean values of ICVF and ODI, as obtained from GM ROIs, are reported in Supplementary Table 6.

Briefly, compared with controls, bvFTD displayed reduced ICVF and ODI in bilateral frontal and anterior temporal cortices, with a slightly greater involvement on the right side. svPPA showed ICVF changes only in left temporal cortex and widespread ODI reductions across both hemispheres relative to controls. nfvPPA showed widespread ICVF (only left hemisphere) and ODI reductions compared with controls. sbvFTD pattern of alterations included ICVF and ODI reductions only in right hemisphere compared with controls.

### Correlations

In FTD groups, significant correlations were found between ICVF, ODI, and FA values across different brain regions and disease severity and/or global cognitive functioning (see Supplementary Table 9). However, only a limited number of correlations survived the Bonferroni correction for the svPPA and nfvPPA subjects (*r* range = −0.76–0.90; *P* range = 0.02–0.05).

### Machine learning

#### SVM models

Different SVM models were trained on different combinations of neuropsychological and MRI data, aiming to achieve the best accuracy score in correctly classifying subjects belonging to the FTD syndromes (bvFTD, svPPA, nfvPPA and sbvFTD). The accuracy score, precision, recall and F_1_-score of different models are reported in Table 2 and Supplementary Table 10 (accuracy range = 53.4–83.6%; precision range = 39.4–84.4%; recall range = 42.1–81.1%; F_1_-score range = 40.3–82.0%).

**Table 2:** Comparative analysis of machine-learning model performance using different feature combinations

Feature combination	Models metrics				Metrics of models following the feature selection			
	Accuracy (%)	Precision (%)	Recall (%)	F_1_-score (%)	Accuracy (%)	Precision (%)	Recall (%)	F_1_-score (%)
FA	60.3	56.2	58.6	57.0	69.9	71.2	66.4	67.5
MD	83.6	84.4	81.3	81.7	84.9	85.3	83.2	83.5
ICVF	76.7	75.5	76.0	75.7	82.2	81.3	82.2	81.7
ODI	68.5	66.1	64.3	65.1	80.8	86.4	74.0	77.2
PSI	53.4	39.4	42.1	40.3	61.6	45.7	50.8	47.7
FA + MD + ICVF + ODI	76.7	74.5	75.4	74.6	94.5	96.5	92.6	94.3
FA + MD + ICVF + PSI	76.7	74.5	75.4	74.6	97.3	97.5	96.5	96.8
FA + MD + ODI + PSI	69.9	67.6	67.5	67.5	97.3	97.5	98.5	97.9
FA + ICVF + ODI + PSI	68.5	67.9	71.0	69.2	98.6	98.7	99.2	98.9
MD + ICVF + ODI + PSI	68.5	66.0	69.6	67.4	95.9	94.7	95.7	95.1
FA + MD + ICVF + ODI + PSI	76.7	74.5	75.4	74.6	94.5	93.9	93.8	93.8

FA, fractional anisotropy; MD, mean diffusivity; ICVF, intracellular fractional volume, ODI, orientation-dispersion index; PSI, neuropsychological data.

When training the models using the features without a selection process, several configurations based on the MD metrics, alone or in conjunction with other features, reached the maximum accuracy of 83.6% (MD, MD + PSI, MD + FA + PSI and MD + ICVF + PSI). The dummy classifiers exhibited lower performance compared with all the machine-learning models: the classifier that randomly selected classes achieved a mean accuracy score of 24.9% over 1000 iterations, whereas predicting the most frequent class (bvFTD) resulted in an accuracy of 45.2%. This substantial performance gap confirms the ability of our models in making meaningful predictions based on information extracted from the data.

#### Sequential features selection

The sequential features selection algorithm allowed to select the most useful variables for the classification and model training. Results are summarized in Table 2 and Supplementary Table 10 (accuracy range = 61.6–98.6%; precision range = 45.7–98.6%; recall range = 50.8–99.2% and F_1_-score range = 47.7–98.9%). After feature selection, the best model was the one trained on a multimodal set including FA, ICVF, ODI and neuropsychological data, reaching an accuracy of 98.6% (Table 2 and Supplementary Table 10). All the features selected by the model are listed in Supplementary Table 11. Results concerning other machine-learning approaches (e.g. random forest, Naive Bayes, XGBoost and logistic regression) revealed that, despite the small sample size, SVM remains the preferable method for our classification analysis (Supplementary Table 12).

#### Model explainability

**Figure 5:**
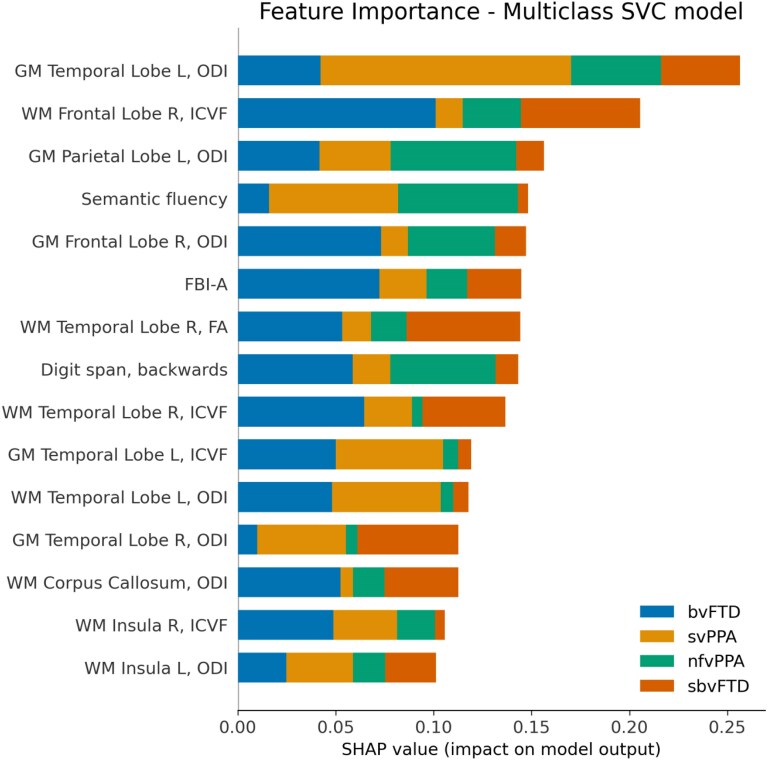
**Multiclass classification. SHAP plot of global feature importance for the 20 most important features.** Predictors are plotted considering their individual composition ratio (different colour bars; scale shown at the bottom of the plot) in descending order of importance. SHAP value is the amount the predictor contributes to the model output. FBI, frontal behavioural inventory; GM, grey matter; ICVF, intracellular fractional volume; L, left; ODI, orientation-dispersion index; R, right; WM, white matter.

The interpretability analysis carried by calculating Shapley values showed the impact of each feature in the model output. The 15 most important features contributing in the SVM model classification are reported in Fig. 5, while the dependency analysis, showing the patient-wise contribution of each feature to the output of the model, is presented in Supplementary Fig. 2.

## Discussion

Our study showed that NODDI is sensitive in detecting both brain GM and WM alterations in subjects across the FTD clinical spectrum, offering advantages over DTI. ICVF, ODI, FA and MD values in various brain regions were associated with disease severity and cognitive function. NODDI also revealed distinct alteration patterns, even from a topographical perspective, within each FTD phenotype, suggesting a close relationship with clinical characteristics, as these vary significantly from phenotype to phenotype. We also demonstrated that leveraging the subtle microstructural changes captured by NODDI and combining them with the computational power of machine learning allowed for an accurate characterization of FTD syndromes. Implementation of SHAP, a model explainability method, enhanced understanding of the model decision process, illustrating the contribution of features and their impact on the classifier’s prediction for each subject. This framework showed promising results, and might be useful in clinical practice, improving accuracy and reliability of differentiating syndromes in the FTD clinical spectrum.

### Diffusion MRI shows extensive microstructural brain alteration in FTD

Using DTI, FA and MD changes were observed in the WM of all FTD groups relative to controls. Those findings are consistent with previous studies demonstrating early WM abnormalities in both bvFTD and PPA syndromes using DTI, with patterns of alterations specific to each syndrome.^7,37^ Furthermore, subjects affected by the recently described sbvFTD syndrome showed a distinct pattern of FA reduction and MD increase primarily localized to frontotemporal regions, with a predominant right temporal lobe involvement. In all the FTD groups, the areas of FA reduction and MD increase extended beyond the areas affected by GM atrophy as identified by VBM when compared with controls, reinforcing the knowledge that early and substantial WM alterations are hallmark features of FTD.^7,38^

In FTD, NODDI metrics reveal unique WM alterations compared with controls, aligning with recent reports of similar results in this group of patients.^21^ ICVF maps show reduced values across all FTD groups, with localized patterns in regions considered the epicentre of the neurodegenerative process in each syndrome.^39^ These alterations align more closely with areas of GM atrophy compared with FA. nfvPPA cases deviate from this pattern, displaying widespread left temporofrontal damage on ICVF maps despite a shorter disease duration and limited GM atrophy clusters.

ICVF is a metric that predominantly reflects the neurite (i.e. axons and dendrites) density within the imaged voxel, a known component of neurodegeneration. Validation studies have demonstrated that ICVF correlates with histologically quantified axonal density measures.^15^ The unique ICVF pattern seen in nfvPPA may be linked to a higher frequency of tau proteinopathy compared with other FTD variants.^40,41^ Indeed, previous studies suggest more severe WM pathology in FTD-tau than FTD-TDP43.^38^ Further investigations with pathological validation are needed to confirm this hypothesis.

ODI maps revealed significant differences in WM microstructure in all FTD groups compared with controls. bvFTD and svPPA showed both reductions and increases in ODI in different WM tracts, while nfvPPA and sbvFTD only showed ODI decreases in specific tracts. ODI quantifies the angular variation or dispersion of neurites within a voxel and reflects neurite coherence, with lower values indicating fibre alignment and higher values denoting greater complexity or less coherence in local neurite orientation. This metric showed correlation with histology-derived index of orientation dispersion (circular variance) in spinal cord samples of controls and multiple sclerosis cases.^42^ Decreased ODI suggests reduced neurite complexity, possibly due to dendritic loss, while increased ODI may be linked to processes like neuroinflammation or dendritic growth. The biological significance of ODI in WM remains unclear, but it likely involves complex interactions in axon alignment and the surrounding environment. Interpreting higher ODI values in bvFTD and svPPA is challenging due to observed bimodal alterations. Notably, reductions in ICVF and ODI increases contribute to FA reduction,^17,43^ reflecting diverse processes in different WM areas—some with reduced neurite density and others with increased ODI.

NODDI excels in studying GM compared with the limitations of DTI. In different patient groups, GM alterations were evident in ICVF and ODI maps, indicating reduced cellular density and changes in cortical architecture. Focal ICVF reductions mirrored WM patterns, while ODI alterations were more widespread, suggesting dendritic and synaptic changes. These findings align with studies in AD, where ICVF reductions precede dementia, while ODI changes indicate later-stage alterations in dendritic architecture.^44^

### Machine learning reveals discriminative multimodal biomarkers for FTD variants

Our study showed NODDI metrics and machine learning effectively differentiate FTD syndromes. Unlike prior research, we used a multimodal approach, integrating features from DTI, NODDI, and neuropsychological data.^28,45-47^ The model trained solely on MD achieved 83.6% accuracy. After feature selection, the top-performing neuroimaging model (MD and ICVF) achieved 91.8% accuracy, highlighting the importance of jointly analysing metrics derived from different diffusion imaging analysis frameworks to effectively capture white and GM characteristics. The best overall model, incorporating FA, ICVF, ODI and neuropsychological features, achieved 98.6% accuracy. Notably, the most contributing features to each syndrome classification were most frequently derived from neuroimaging data. Indeed, the observed alterations in FA, ICVF and ODI underscore the complex nature of FTD pathophysiology, reflecting a combination of neuronal loss, dendritic remodelling and microglial activation. These changes likely contribute to the diverse clinical presentations observed across FTD subtypes, highlighting the importance of multimodal imaging approaches in elucidating underlying disease mechanisms. It is important to note that neuropsychological data remarkably contributed to model performance. Higher frontal behavioural inventory (FBI)-A scores increased the likelihood of bvFTD, indicating negative behavioural symptoms,^48^ while lower FBI-A scores suggested sbvFTD diagnosis. This may be due to the fact that sbvFTD patients do not exhibit the classic negative symptoms of bvFTD described and assessed by the FBI-A. Instead, they present altered socioemotional behaviours, such as changed emotional expression, social reactions, motivation for social interactions and altered prioritization, including hyperfocus on specific interests and altered hedonic valuation and personal preferences. These symptoms, recently suggested by the Consensus Recommendations of the International Working Group, are not captured by standard questionnaires like the FBI.^49^ Semantic fluency deficits strongly suggested svPPA and poorly suggested nfvPPA, aligning with expectations.^3^ Poor and high digit span backward performances contributed to nfvPPA and bvFTD classification, respectively, consistent with known language production differences.^50^ Conversely, no cognitive features contributed to sbvFTD identification, reflecting its complexity beyond standard testing domains.^4^ Given the relatively low number of patients included, these findings should be interpreted with caution, and further validation on larger cohorts is warranted.

### Limitations and conclusions

Our study had limitations. First, the lack of pathological confirmation of FTD diagnoses; although, when available, cerebrospinal fluid results were not suggestive for AD, further investigations using post-mortem pathology assessments are necessary. Second, the sample is relatively small and we lack an external validation dataset. Notably, acquiring an external validation dataset can indeed pose challenges in the context of rare diseases such as FTD and its subtypes, primarily due to the rarity of the condition and the difficulty in obtaining MRI scans. Although larger MRI datasets of FTD patients are available, the absence of advanced imaging techniques such as multi-shell diffusion imaging used in this study limit their use in such studies. To overcome this limitation, cross-fold validation was used. Third, the machine learning section was hindered by imbalanced group sizes, due to the difficulty in enrolling rare FTD syndromes. The uneven distribution of subjects among different FTD syndromes may have introduced bias and affected the generalizability of our model’s performance. To overcome this limitation, future studies should strive to include a more balanced and representative sample. Fourth, it is essential to acknowledge that this study employed a cross-sectional design, which inherently limits our ability to track disease progression.

## Conclusion

Our study has shed the light on the application of NODDI-derived metrics and machine-learning algorithms for characterizing FTD. NODDI effectively captures subtle microstructural alterations in both GM and WM, presenting advantages over standard DTI. Integrating the rich information from NODDI with cognitive data enables machine-learning models to learn complex patterns and relationships among various FTD subtypes. The combined use of NODDI and machine-learning algorithm holds promise for enhancing our understanding of FTD pathology and facilitating individual-level clinical differentiation.

## Supplementary Material

fcaf442_Supplementary_Data

## Data Availability

The anonymized dataset used and analysed during the current study will be made available by the corresponding author upon request to qualified researchers (i.e. affiliated to a university or research institution/hospital). The codes that support the findings of this study are openly available in GitHub public repository at https://github.com/SilviaBasaia/Frontotemporal-dementia-characterization-using-NODDI.
