# Supplementary material for: Frontotemporal dementia characterization using neurite orientation dispersion and density imaging
Source: Brain Commun. 2025 Nov 11;7(6):fcaf442. doi: 10.1093/braincomms/fcaf442 (PMC12673379; doi:10.1093/braincomms/fcaf442)
Supplement: fcaf442_Supplementary_Data [file fcaf442_supplementary_data.pdf]

SUPPLEMENTARY MATERIALS

Summary: Detailed description of methods for neurophychological assessment, MRI acquisition and analysis, and relative results. Includes 10 supplementary tables and 1 supplementary figure.

File type: .docx

MATERIALS AND METHODS

Neuropsychological assessment

The neuropsychological assessment was performed by experienced neuropsychologists, unaware of MRI results. The following domains were investigated: global cognitive functioning with the Mini Mental State Examination (MMSE)[1] and the frontal assessment battery (FAB);[2] long and short term verbal memory with the Rey Auditory Verbal Learning Test[3] and the digit span forward,[4] respectively; long and short term visuospatial memory with the recall of the Rey's complex figure[5] and the spatial span forward;[4] attention and executive functions with the digit span backward,[6] Raven's colored progressive matrices (RCPM),[7] Trail Making Test,[8] attentive matrices[9] and the Modified Card Sorting Test;[10] social cognition with the intention and emotion attribution of the Story-based Empathy Task (SET),[11] and the subtests (affect discrimination, affect naming, affect matching and affect selection) of the Comprehensive Affect Testing System (CATS);[12] language with the token test,[13] and with the semantic and phonemic fluencies;[14] visuospatial abilities with the Clock Drawing Test (CDT),[15] and the copy of Rey's complex figure[5] and of drawings without landmarks;[3] praxis was tested with orofacial ideomotor apraxia test,[9] and with the Goldenberg's test;[16] the presence of behavioral disturbances with the A and B sections of the frontal behavioral inventory (FBI)[17] administered to patients' caregivers. The healthy controls were administered with the same neuropsychological assessment except for FAB, CDT, SET, orofacial and Goldenberg ideomotor apraxia, FBI.

For the machine learning analysis, the following neuropsychological variables were selected: FAB, RCPM, token test, phonemic and semantic fluency, digit span backward, SET (emotion attribution), FBI A and B.

MRI acquisition parameters

Using a 3.0 T scanner (Ingenia CX, Philips), the following brain MRI sequences were obtained from all participants: 3D T1-weighted turbo field echo (TFE) (TR=7 ms; TE=3.2 ms; flip angle=9 [degrees]; 204 contiguous sagittal slices with voxel size=1 x 1 x 1 mm, matrix size=256 x 240, FOV=256x240 mm^2); 3D FLAIR (TR=4800 ms; TE=267 ms; TI=1650 ms; ETL=167; NEX=2; 192 contiguous sagittal slices with voxel size=0.89 x 0.89 x 1 mm, matrix size=256 x 256, FOV=256x256 mm2); 3D T2 (TR=2500 ms; TE=330 ms; ETL=117; NEX=1; 192 contiguous sagittal slices with voxel size=0.89 x 0.89 x 1 mm, matrix size=256 x 258, FOV=256x256 mm2); and axial pulsed-gradient spin echo (PGSE) single shot DW EPI sequence: shells at b value = 700/1000/2855 s/mm2 along 6/30/60 non-collinear directions, 10 b = 0 volumes were acquired distributed along the acquisition and three additional b=0 s/mm^2 volumes with reversed polarity of gradients for distortion correction were acquired with a separate sequence (TR=5900 ms, TE=78 ms, voxel size = 2.14 × 2.69 mm, 56 slices, 2.3 mm thick, matrix size=112×85, FOV = 240 × 233 mm^2).

VBM Pre-processing

Voxel-based morphometry (VBM) was performed using SPM12 (http://www.fil.ion.ucl.ac.uk/spm/) and Diffeomorphic Anatomical Registration Exponentiated Lie Algebra (DARTEL) registration method, to detect GM volume alterations. Briefly, (i) T1-weighted images were segmented to produce GM, WM and CSF tissue probability maps in the MNI space; (ii) the segmentation parameters obtained from the step (i) were imported in DARTEL; (iii) the rigidly aligned version of the images previously segmented (i) was generated; (iv) the DARTEL template was created and the

obtained flow fields were applied to the rigidly-aligned segments to warp them to the common DARTEL space and then modulated using the Jacobian determinants. Since the DARTEL process warps to a common space that is smaller than the MNI space, we performed an additional transformation as follows: (v) the modulated images from DARTEL were normalized to the MNI template using an affine transformation estimated from the DARTEL GM template and the a priori GM probability map without resampling (http://brainmap.wisc.edu/normalizeDARTELtoMNI). Prior to statistical computations, images were smoothed with an 8 mm FWHM Gaussian filter.

Diffusion weighted MRI Pre-processing

Preprocessing of diffusion-weighted data included skull-stripping and correction for head motions by aligning the volumes to the first B0 volume, as well as susceptibility-induced field and eddy currents distortion correction, using the tools implemented in the FMRIB software library (FSL, version 5.0.9).

TBSS processing

For each subject, FA and MD maps obtained from the previous DWI processing steps were aligned to the FMRIB58 template included in FSL, using non-linear registration (fsl FNIRT tools). A mean FA image was calculated, averaging all the aligned images in the common space, and the result was thinned to obtain a skeleton representing only the white matter tracts common to all the subjects. The FA skeletonized maps were thresholded to exclude voxels below the intensity of 0.2. The FA maps of each individual were projected to this skeleton mask. MD, ICVF and ODI were also projected to the same skeleton mask, using the tbss_non_FA tool in FSL.

GBSS processing

T1, T2 and DWI scans of each patient were registered through linear and non-linear transformation to the MNI152 space (2 mm resolution), using FSL FLIRT and FNIRT tools.

The resulting warping matrices were subsequently applied to the GM probability maps (obtained from VBM segmentation) and NODDI-derived ICVF and ODI maps, to align all images to the MNI152 common space. An average gray matter probability map was calculated using fslmaths and skeletonized. For each patient, local voxels with the greatest gray matter probability were projected to the GM skeleton. The gray matter skeletonized map was thresholded, keeping voxels higher than 0.65 in more than 75% of the subjects. For each subject, individual diffusion metrics from ICVF and ODI maps were projected on the thresholded skeleton from the voxels with the greatest gray matter probability obtained from the previous step. The remaining missing voxels from the skeleton mask were filled with the average of the surrounding voxels on the skeleton, weighted by closeness with a Gaussian Kernel (σ=2 mm).

Machine learning

A classifier model, trained on MRI and neuropsychological features, was constructed to identify hyperplanes which maximize the distance between subjects belonging to different classes (different clinical FTD syndromes). Healthy controls were not included in such analysis, as we aimed to provide a model useful in differentiating the clinical syndromes across the FTD spectrum. A multiclass, one-vs-rest, support vector machine (SVM) classification algorithm was used, with a linear kernel (tuning cost parameter = 0.25, hinge loss function and l2 penalty, tolerance for the stopping function of 1e-4, and 1000 maximum number of iterations). Machine learning analysis was implemented in Python 3.10.

Multiple classifier models were trained on different combinations of 76 MRI features (mean FA, MD, ICVF and ODI values of the USCLobes ROIs) and 9 selected neuropsychological tests (see Supplementary materials for details). A set of standard neuropsychological tests and/or clinical questionnaires was selected assessing the cognitive and behavioural domains known to be the most associated with alterations in the FTD spectrum: attention and executive functions, social cognition, fluency, and behaviour. Among these tests/questionnaires, to ensure data quality, we further

selected only those that were obtained in at least 60% of patients. For the remaining missing values, we employed a robust imputation method using the median of the corresponding group, aiming to minimize potential biases. The final set of the selected neuropsychological variables are reported in the Neuropsychological assessment section. All the 76 features were standardized prior to training to obtain a unit normal distribution with a mean equal to zero and a variance to 1.

RESULTS

VBM

Compared to controls, all disease groups exhibited GM atrophy. bvFTD patients demonstrated widespread frontotemporal GM atrophy involving frontal and temporal lobes, insula, and anterior and middle cingulate cortices bilaterally. In contrast, svPPA patients revealed asymmetric GM atrophy with a more pronounced damage on the left side, involving temporal and orbitofrontal cortices relative to controls. nfvPPA cases showed focal atrophy localized to the left hemisphere with clusters in frontal, parietal and temporal cortex. Despite the small number of patients, sbvFTD group exhibited significant GM atrophy, predominantly asymmetrical, with a right predominant involvement of bilateral temporal lobe and extending into the ipsilateral medial orbitofrontal cortex relative to controls.

TBSS

bvFTD vs others

bvFTD patients exhibited a widespread pattern of decreased FA and increased MD relative to controls. FA reduction and increased MD was also observed relative to svPPA and nfvPPA cases in fronto-temporal fibers, with right predominant involvement. In addition, bvFTD patients showed reduced FA and increased MD restricted to frontal areas bilaterally when compared to sbvFTD.

bvFTD patients relative to controls were characterized by a bilateral frontotemporal pattern of ICVF alterations, more focal compared with that of FA. ICVF reduction was also found in bvFTD when compared to the other patient groups. Bilateral fronto-temporal tracts were affected in comparison to svPPA and sbvFTD, while compared to nfvPPA the involvement was primarily in right frontotemporal fibers.

bvFTD patients showed a significant reduction of ODI relative to controls in bilateral corona radiata, internal capsule, corpus callosum, and the superior longitudinal fasciculus. On the other hand, some areas in bvFTD showed an increased ODI relative to controls, primarily involving the

parieto-occipital bundles with a slight left-sided predominance. Compared to other FTD cases, bvFTD showed increased ODI in inferior longitudinal fasciculus relative to svPPA patients.

svPPA vs others

svPPA patients showed reduced FA and increased MD in a wide range of fibers when compared to controls, displaying a fronto-temporal pattern with predominant involvement of the left side. In addition, increased MD was found in svPPA relative to bvFTD and nfvPPA cases, primarily involving the left temporal fibers, sagittal stratum, posterior thalamic radiation, and uncinate fasciculus.

Compared to controls, ICVF maps showed alterations in svPPA, similar to FA but more focal, primarily involving left fronto-temporal regions (i.e., anterior corona radiata, external capsule, inferior and superior longitudinal fasciculus, cingulate fibers and cerebral peduncle).

SvPPA patients exhibited reduced ODI in different regions compared to controls: anterior and superior corona radiata, corpus callosum, internal capsule, left inferior longitudinal fasciculus. An increased value of ODI was found in the left stria terminalis tracts compared to controls. In addition, svPPA group showed reduced ODI in left temporal fibers compared to bvFTD and nfvPPA, and in corpus callosum and left superior corona radiata compared to sbvFTD.

nfvPPA vs others

Comparing nfvPPA patients to controls, FA and MD maps revealed alterations involving fronto-temporal WM fiber bundles with a left-sided prevalence. In addition, increased MD was found in nfvPPA relative to sbvFTD cases, primarily involving fronto-parietal white matter tracts.

Similarly, the ICVF maps of nfvPPA patients displayed a broad involvement of frontotemporal fibers, with reduced values primarily on the left side when compared to controls. ICVF affected areas overlapped with those identified in the FA map with a greater involvement of bilateral internal and external capsules (with left side prevalence), and posterior extension to the left

posterior corona radiata and posterior thalamic radiation. Moreover, nfvPPA group showed ICVF reduction in the body of corpus callosum and left frontal fibers compared to svPPA, and in the left frontal and parietal fibers compared to sbvFTD. ODI maps showed a reduction in nfvPPA compared to controls, involving bilateral corona radiata, corpus callosum, internal capsule, cerebral peduncles and corticospinal tract, as well as the left superior longitudinal fasciculus. Compared to sbvFTD group, ODI decrease was observed in left corona radiate in nfvPPA patients.

sbvFTD vs others

FA and MD maps showed a significant alteration in sbvFTD patients relative to controls primarily in temporal, but also in frontal fibers, with greater involvement of the right side. When compared to bvFTD, svPPA, and nfvPPA, sbvFTD group showed FA decrease involving right temporal fibers and external capsule.

Comparing sbvFTD patients to controls, ICVF maps demonstrated an involvement similar to FA, but more focused on the right side, affecting inferior frontal and temporal regions. Compared to nfvPPA, sbvFTD patients showed ICVF reduction involving right inferior longitudinal fasciculus and external capsule.

ODI was decreased in right corona radiata, as well as in the superior longitudinal fasciculus in sbvFTD patients compared to controls.

GBSS

bvFTD vs others

Decreased ICVF was found in bvFTD patients compared to controls, involving bilateral frontal and anterior temporal cortices, sparing the hippocampal and parahippocampal region, with a slight greater involvement of the right side. Additionally, bilateral putamen and caudate nuclei showed reduced ICVF values. When comparing bvFTD to svPPA cases, reduced ICVF was observed, involving right superior frontal gyrus, inferior orbital cortex and insula.

ODI maps showed widespread GM alterations in bvFTD patients relative to controls. In addition, reduced ODI involved right frontal lobe and insula when compared to svPPA and nfvPPA patients. Furthermore, right caudate and putamen ODI values were reduced compared to nfvPPA. Compared to sbvFTD, bvFTD patients showed reduced ODI in bilateral superior frontal cortex, extending to the left postcentral gyrus.

svPPA vs others

When comparing svPPA to controls, decreased ICVF was found only in the left hemisphere, involving the temporal cortex and small areas of anterior cingulum and left caudate.

In contrast, ODI maps showed widespread reduced values in svPPA patients relative to controls, with an asymmetrical involvement. On the left side, ODI reduction encompassed frontal, temporal and parietal lobes, with a small involvement of occipital cortex. On the right side, decreased ODI involved orbitofrontal and superior frontal cortex, supramarginal and postcentral gyri, superior parietal lobe, and right caudate. svPPA showed reduced ODI involving left amygdala and hippocampus when compared to bvFTD and nfvPPA cases; additionally, compared to nfvPPA, ODI reduction was also present in anterior temporal lobe and parahippocampal regions.

nfvPPA vs others

Compared to controls, nfvPPA group showed reduced ICVF in the left hemisphere, involving frontal, temporal and anterior parietal cortices. In addition, nfvPPA patients displayed a reduction in middle occipital cortex compared to bvFTD group, and a reduction in superior frontal cortex compared to svPPA.

ODI maps revealed a bilateral decrease with left-predominant pattern in nfvPPA patients compared to controls. The alteration involved bilateral frontal, temporal and parietal lobes, with extension to the left occipital lobe. Furthermore, the nfvPPA group exhibited reduced ODI values when compared to sbvFTD, involving the left superior frontal and parietal cortices.

sbvFTD vs others

sbvFTD group showed areas of focal ICVF reduction in right hemisphere when compared to controls, involving insula, olfactory, temporal and inferior occipital cortices.

ODI maps revealed a greater pattern of reduced values when comparing sbvFTD to controls but still in the right hemisphere, with involvement of insula, temporal frontal and parietal lobes, middle occipital and cingulate cortex. Additionally, compared to nfvPPA, sbvFTD showed a small cluster of ODI reduction in right amygdala and parahippocampal regions.

REFERENCES

1. Folstein MF, Folstein SE, McHugh PR. "Mini-mental state". A practical method for grading the cognitive state of patients for the clinician. *J Psychiatr Res*. Nov 1975;12(3):189-98. doi:10.1016/0022-3956(75)90026-6
2. Appollonio I, Leone M, Isella V, et al. The Frontal Assessment Battery (FAB): normative values in an Italian population sample. *Neurological sciences : official journal of the Italian Neurological Society and of the Italian Society of Clinical Neurophysiology*. Jun 2005;26(2):108-16. doi:10.1007/s10072-005-0443-4
3. Carlesimo GA, Caltagirone C, Gainotti G. The Mental Deterioration Battery: normative data, diagnostic reliability and qualitative analyses of cognitive impairment. The Group for the Standardization of the Mental Deterioration Battery. *Eur Neurol*. 1996;36(6):378-84. doi:10.1159/000117297
4. Orsini A, Grossi D, Capitani E, Laiacona M, Papagno C, Vallar G. Verbal and spatial immediate memory span: normative data from 1355 adults and 1112 children. *Italian journal of neurological sciences*. Dec 1987;8(6):539-48. doi:10.1007/bf02333660
5. Caffarra P, Vezzadini G, Dieci F, Zonato F, Venneri A. Rey-Osterrieth complex figure: normative values in an Italian population sample. *Neurol Sci*. Mar 2002;22(6):443-7. doi:10.1007/s100720200003
6. Monaco M, Costa A, Caltagirone C, Carlesimo GA. Forward and backward span for verbal and visuo-spatial data: standardization and normative data from an Italian adult population. *Neurol Sci*. May 2013;34(5):749-54. doi:10.1007/s10072-012-1130-x
7. Basso A, Capitani E, Laiacona M. Raven's coloured progressive matrices: normative values on 305 adult normal controls. *Funct Neurol*. Apr-Jun 1987;2(2):189-94.
8. Giovagnoli AR, Del Pesce M, Mascheroni S, Simoncelli M, Laiacona M, Capitani E. Trail making test: normative values from 287 normal adult controls. *Ital J Neurol Sci*. Aug 1996;17(4):305-9. doi:10.1007/BF01997792
9. Spinnler H, Tognoni, G. . Standardizzazione e taratura italiana di test neuropsicologici. *Ital J Neurol Sci* 1987;6(8):44-46.
10. Caffarra P, Vezzadini G, Dieci F, Zonato F, Venneri A. Modified Card Sorting Test: normative data. *J Clin Exp Neuropsychol*. Apr 2004;26(2):246-50. doi:10.1076/jcen.26.2.246.28087
11. Dodich A, Cerami C, Canessa N, et al. A novel task assessing intention and emotion attribution: Italian standardization and normative data of the Story-based Empathy Task. *Neurol Sci*. Oct 2015;36(10):1907-12. doi:10.1007/s10072-015-2281-3
12. Schaffer SG, Wisniewski A, Dahdah M, Froming KB. The comprehensive affect testing system-abbreviated: effects of age on performance. *Arch Clin Neuropsychol*. Feb 2009;24(1):89-104. doi:10.1093/arclin/acp012
13. De Renzi E, Vignolo LA. The token test: A sensitive test to detect receptive disturbances in aphasics. *Brain*. Dec 1962;85:665-78. doi:10.1093/brain/85.4.665
14. Novelli G LM, Papagno C, Vallar G, Capitani E, Cappa SF. . Three clinical tests to research and rate the lexical performance of normal subjects. . *Arch Psicol Neurol Psichiatr* 1986;47:477–506.
15. Manos PJ. Ten-point clock test sensitivity for Alzheimer's disease in patients with MMSE scores greater than 23. *Int J Geriatr Psychiatry*. Jun 1999;14(6):454-8.
16. Goldenberg G. Neuropsychological assessment and treatment of disorders of voluntary movement. . In: Halligan K, & Marshall ed. *Handbook of clinical neuropsychology* 2003.
17. Alberici A, Geroldi C, Cotelli M, et al. The Frontal Behavioural Inventory (Italian version) differentiates frontotemporal lobar degeneration variants from Alzheimer's disease. *Neurol Sci*. Apr 2007;28(2):80-6. doi:10.1007/s10072-007-0791-3

Supplementary Table 1. Cognitive and behavioral characteristics of the sample.

Variables	HC	bvFTD	svPPA	nfvPPA	sbvFTD
**Number**	48	35	20	14	9
Global cognition					
**MMSE (9/126)**	29.25±0.87$^{\blacklozenge\,\#}$ (27.00-30.00)	23.57±5.54* (6.00-30.00)	21.58±7.62* (5.00-30.00)	24.17±7.82 (5.00-30.00)	24.87±4.61 (18.00-30.00)
**FAB (14/78)**	-	11.32±4.01 (3.00-17.00)	11.94±4.72 (0.00-17.00)	12.30±3.40 (5.00-17.00)	13.12±1.46 (11.00-15.00)
Memory					
**Digit span, forward (12/126)**	5.91 ± 1.00§ (4.00-8.00)	5.00±1.54 (0.00-7.00)	5.00±1.20 (2.00-7.00)	4.09±0.94* (2.00-5.00)	5.62±0.51 (5.00-6.00)
**RAVLT, delayed recall (18/126)**	10.66±2.29$^{\blacklozenge\,\#\,\S\,\text{※}}$ (6.00-15.00)	2.44±2.66$^{*\,\S}$ (0.00-8.00)	3.27±3.26$^{*\,\S}$ (0.00-10.00)	7.10±3.45$^{*\,\blacklozenge\,\#}$ (0.00-12.00)	3.87±3.27* (0.00-10.00)
**Spatial span, forward (18/126)**	5.35±1.1 (3.00-7.00)	4.25±1.39 (2.00-7.00)	4.41±1.28 (2.00-7.00)	4.36±1.29 (2.00-6.00)	4.89±0.93 (4.00-7.00)
**Rey's complex figure, recall (43/126)**	14.75±5.76$^{\blacklozenge}$ (6.50-24.00)	7.29±6.19* (0.00-27.50)	9.97±8.03 (0.00-25.00)	9.60±4.91 (4.50-16.50)	8.25±7.32 (0.00-18.50)
Attention and executive function					
**Attentive matrices (15/126)**	51.93±6.34$^{\blacklozenge\,\#\,\S}$ (27.00-60.00)	41.38±11.14* (22.00-60.00)	39.42±15.45* (12.00-56.00)	38.18±15.59* (7.00-54.00)	50.37±5.65 (43.00-59.00)
**RCPM (19/126)**	32.08±3.42$^{\blacklozenge\,\#}$ (17.00-36.00)	21.44±7.77* (8.00-35.00)	24.29±9.65* (3.00-36.00)	25.44±8.89 (6.00-34.00)	24.87±4.22 (20.00-32.00)
**Digit span, backward (16/126)**	4.91±1.24$^{\blacklozenge\,\#\,\S}$ (3.00-8.00)	3.26±1.23* (0.00-5.00)	3.17±1.46* (0.00-5.00)	2.73±0.79* (2.00-4.00)	4.14±0.69 (3.00-5.00)
**MCST, perseverations (32/126)**	3.69±3.74$^{\blacklozenge}$ (0.00-16.00)	16.90±12.12$^{*\,\#\,\text{※}}$ (1.00-44.00)	4.50±4.22$^{\blacklozenge}$ (0.00-13.00)	9.125±9.66 (0.00-25.00)	5.17±7.49$^{\blacklozenge}$ (0.00-20.00)
**TMT, B-A (36/126)**	63.64 ± 38.45$^{\blacklozenge}$ (19.99-209.69)	199.90±141.13$^{*\,\text{※}}$ (67.00-617.85)	115.24±78.70 (26.00-340.00)	108.69±39.94 (55.00-179.53)	101.90±35.86$^{\blacklozenge}$ (43.00-147.00)
Visuospatial abilities					

**Rey's complex figure copy (42/126)**	29.07±3.74 (21.00-35.00)	25.59±9.21 (0.00-36.00)	29.03±6.50 (14.00-35.00)	24.25±12.08 (0.00-34.00)	30.69±3.03 (25.00-35.00)
**Copy of drawings without landmarks (22/126)**	10.38±1.50[§] (5.00-12.00)	9.54±2.08 (3.00-12.00)	10.12±1.54 (8.00-12.00)	8.40±2.67[*] (2.00-11.00)	9.87±0.83 (9.00-11.00)
**Ten-point clock test (12/78)**	-	5.28±3.83 (0.00-10.00)	4.83±4.15 (0.00-10.00)	6.54±4.06 (0.00-10.00)	6.25±3.69 (0.00-10.00)
Language & fluency					
**Token test (19/126)**	34.51±1.34[♦ #] (31.00-36.00)	26.28±7.95[*] (5.00-35.00)	26.06±10.06[*] (4.00-36.00)	27.19±5.93 (14.00-32.00)	29.25±5.28 (19.50-35.00)
**Phonemic fluency (13/126)**	37.79±8.50[♦ # § ※] (23.00-59.00)	17.18±11.33[*] (0.00-47.00)	18.67±10.44[*] (0.00-31.00)	11.36±7.83[*] (1.00-25.00)	18.12±7.55[*] (7.00-33.00)
**Semantic fluency (13/126)**	47.64±9.52[♦ # § ※] (27.00-70.00)	19.54±7.25[*#] (3.00-30.00)	11.50±7.20 [* ♦ §] (0.00-23.00)	22.09±11.85[* #] (0.00-36.00)	21.37±12.21[*] (0.00-36.00)
Social cognition					
**CATS, affect discrimination (29/126)**	11.26±0.74[♦] (10.00-12.00)	9.79±1.84[*] (6.00-12.00)	10.43±1.50 (8.00-12.00)	10.44±1.13 (9.00-12.00)	11.25±1.16 (9.00-12.00)
**CATS, affect naming (29/126)**	4.58±1.07[♦ # § ※] (2.00-6.00)	2.56±1.24[*] (0.00-4.00)	3.29±1.90[*] (1.00-6.00)	3.22±1.48[*] (1.00-6.00)	2.50±1.07[*] (1.00-4.00)
**CATS, affect matching (31/126)**	8.98±1.93[♦ # § ※] (5.00-12.00)	6.18±1.68[*] (3.00-9.00)	7.36±2.13[*] (4.00-11.00)	6.11±2.85[*] (0.00-9.00)	5.62±0.92[*] (4.00-7.00)
**CATS, affect selection (30/126)**	5.58±0.71[♦ # § ※] (3.00-6.00)	3.78±1.20[*] (2.00-6.00)	4.43±1.28[*] (2.00-6.00)	4.33±1.58[*] (2.00-6.00)	3.125±0.99[*] (2.00-4.00)
**SET, intention attribution (27/78)**	-	3.52±1.47 (1.00-6.00)	3.83±1.95 (0.00-6.00)	3.80±1.55 (1.00-6.00)	2.00±1.00 (0.00-3.00)
**SET, emotion attribution (27/78)**	-	3.33±1.35 (1.00-6.00)	3.42±2.11 (0.00-6.00)	3.90±2.02 (0.00-6.00)	3.29±1.80 (1.00-6.00)
Praxia					
**Goldenberg's test, limb ideomotor apraxia**	-	32.54±9.39 (0.00-40.00)	35.87±4.13 (28.00-40.00)	32.44±7.67 (14.00-39.00)	37.75±2.96 (33.00-40.00)

**(23/78)**					
**Buccofacial ideomotor apraxia (22/78)**	-	17.70±3.34 (9.00-20.00)	17.37±3.96 (8.00-20.00)	16.90±2.33 (13.00-20.00)	18.16±2.79 (13.00-20.00)
Mood & Behaviour					
**FBI, total (22/78)**	-	23.00±11.36 (6.00-51.00)	18.07±12.42 (6.00-40.00)	13.00±9.96 (2.00-36.00)	22.12±9.48 (11.00-37.00)
**FBI, A (22/78)**	-	14.13±6.50 (4.00-27.00)	11.57±8.20 (2.00-28.00)	9.50±6.30 (2.00-20.00)	13.25±4.46 (8.00-20.00)
**FBI, B (22/78)**	-	8.86±7.43 (1.00-24.00)	6.50±5.75 (0.00-20.00)	3.50±4.68 (0.00-16.00)	8.87±6.15 (2.00-17.00)

Values are means ± standard deviations [range]. *p* values refer to ANOVA models, corrected for age, sex and education, followed by post-hoc pairwise comparisons, Bonferroni-corrected for multiple comparisons. The threshold of statistical significance was set at p<0.05. * = statistically significant difference with HC; ♦ = statistically significant difference with bvFTD; # = statistically significant difference with svPPA; § = statistically significant difference with nfvPPA; ※=statistically significant difference with sbvFTD. Abbreviations: bvFTD = behavioral variant Frontotemporal Dementia; CATS = comprehensive affect testing system; FAB = Frontal assessment battery; FBI = Frontal behavioural inventory; MCST = modified card sorting test; MMSE = Mini-mental-state evaluation; nfvPPA = nonfluent/agrammatic variant Primary Progressive Aphasia; RAVLT=Rey Auditory Verbal Learning Test; RCPM = Raven's Colored Progressive Matrices; sbvFTD = semantic behavioral variant Frontotemporal Dementia; SET = Story-based empathy task; svPPA = semantic variant Primary Progressive Aphasia; TMT = Trail-making test.

Supplementary Table 2. Results of TBSS analysis on FA maps.

FA maps	Controls	bvFTD	svPPA	nfvPPA	sbvFTD
**Controls**	/	Controls>bvFTD: **B**: Widespread reduction involving all major tracts, including corticospinal, cerebellum and brainstem tracts	Controls>svPPA: **B**: ant. corpus callosum, corona radiata, cerebral peduncles, fornix, external capsule (L>R), cingulate fibres (left more than right), inf. longitudinal fasciculus (L>R) **L:** sup. longitudinal fasciculus, post. thalamic radiation	Controls>nfvPPA: **B:** body and genu of corpus callosum, ant. corona radiata (L>R), superior longitudinal fasciculus (L>R), **L:** external capsule, inf. longitudinal fasciculus, and uncinate fasciculus.	Controls>sbvFTD: B: frontotemporal reduction with right predominant involvement of genu and splenium of corpus callosum, ant. corona radiata, external capsule, inf. longitudinal fasciculus, uncinate, cingulum, posterior thalamic radiations.
**bvFTD**	bvFTD >Controls: none	/	bvFTD>svPPA: none	bvFTD>nfvPPA: none	bvFTD>sbvFTD: none
**svPPA**	svPPA>Controls: none	svPPA>bvFTD: **B:** corpus callosum, ant. and sup. corona radiata, internal capsule, **R:** external capsule, stria terminalis, sup. and inf. longitudinal fasciculus	/	svPPA>nfvPPA: none	svPPA>sbvFTD: none
**nfvPPA**	nfvPPA>Controls: none	nfvPPA>bvFTD: **B:** genu and splenium of corpus callosum, ant. corona radiata, post. thalamic radiation (L>R), sup. longitudinal fasciculus (R>L), corticospinal tract **R:** internal and external capsule, inf. longitudinal fasciculus, uncinate and	nfvPPA>svPPA:none	/	nfvPPA>sbvFTD: **R:** external capsule, stria terminalis, post. thalamic radiation, cingulum, uncinate fasciculum

		cingulate fasciculus, cerebral peduncle			
**sbvFTD**	sbvFTD>Controls:none	sbvFTD>bvFTD:<br>**B:** genu of corpus callosum, ant. corona radiata (L>R),<br>**L:** external capsule	sbvFTD>svPPA:none	sbvFTD>nfvPPA:none	/

Abbreviations: ant.= anterior; B= bilateral; inf.= inferior; L= left; post.= posterior; R= right; sup.= superior.

Supplementary Table 3. Results of TBSS analysis on MD maps.

MD maps	Controls	bvFTD	svPPA	nfvPPA	sbvFTD
**Controls**	/	Controls>bvFTD: None	Controls>svPPA: None	Controls>nfvPPA: None	HC>sbvFTD: none
**bvFTD**	bvFTD>controls: Widespread bilateral WM damage with relative occipital fibers sparing	/	bvFTD>svPPA: B: corona radiata with more post. involvement to the R, superior longitudinal fasciculum (R>L), R external capsule	bvFTD>nfvPPA: B: ant. corpus callosum R: internal and external capsule, anterior corona radiata, superior longitudinal fasciculus	bvFTD>sbvFTD: B: corona radiata and internal capsule L: superior longitudinal fasciculus, external capsule.
**svPPA**	svPPA>Controls: B: callosum, corona radiata, external capsule, posterio thalamic radiation L: internal capsule (post. limb), superior longitudinal fasciculum	svPPA>bvFTD: L: temporal fibers, saggital stratum, posterior thalamic radiation, uncinate fasciculum	/	svPPA>nfvPPA: L: cingulus, saggital stratus, posterior thalamic radiation, uncinate fasciculus	svPPA>sbvFTD: None
**nfvPPA**	nfvPPA>Controls: B: corona radiata, external capsule, superior longitudinal fasciculus, L: internal capsule	nfvPPA>bvFTD: None	nfvPPA>svPPA: None	/	nfvPPA>sbvFTD: L: superior longitudinal fasciculus, internal and external capsule, superior corona radiata
**sbvFTD**	sbvFTD>HC: B: bilateral cingulum R: external capsule, post. thalamic radiation, saggital stratum	sbvFTD>bvFTD: R: temporal lobe fibers	sbvFTD>svPPA: R: uncinate fasciculus, external capsule, saggital stratus	sbvFTD>nfvPPA: R: external capsule, saggital stratum, cingulum, uncinate fasciculum, superior longitudinal fasciculum	/

Abbreviations: ant.= anterior; B= bilateral; inf.= inferior; L= left; post.= posterior; R= right; sup.= superior.

Supplementary Table 4. Results of TBSS analysis on ICVF maps.

ICVF maps	Controls	bvFTD	svPPA	nfvPPA	sbvFTD
**Controls**	/	Controls>bvFTD:<br>B: corpus callosum, corona radiata, internal and external capsule, inf. and sup. longitudinal fasciculus, cingulum, stria terminalis, cerebral peduncles, corticospinal tracts, cerebellar bundles	Controls>svPPA:<br>L: ant. corona radiata, external capsule, inf. and sup. longitudinal fasciculus, stria terminalis, cingulate fibres, cerebral peduncle	Controls>nfvPPA:<br>B: ant. and sup. corona radiata (L>R), genu and body of corpus callosum, internal and external capsule (L>R)<br>L: sup. longitudinal fasciculus, splenium of corpus callosum, post. corona radiata, post. thalamic radiation	Controls>sbvFTD:<br>R: external capsule, inf. longitudinal fasciculus, stria terminalis, cerebral peduncle, ant. corona radiata
**bvFTD**	bvFTD>controls: none	/	bvFTD>svPPA: none	bvFTD>nfvPPA: none	bvFTD>sbvFTD: none
**svPPA**	svPPA>controls: none	svPPA>bvFTD:<br>B: ant. corona radiata, body and genu of corpus callosum, internal capsule, cerebral peduncle, sup. longitudinal fasciculus (R>L)<br>R: external capsule, cingulate	/	svPPA>nfvPPA:<br>B: body of corpus callosum<br>L: ant. corona radiata, internal capsule, sup. longitudinal fasciculus	svPPA>sbvFTD: none
**nfvPPA**	nfvPPA>controls:none	nfvPPA>bvFTD:<br>R: ant. corona radiata, external capsule, inferior longitudinal fasciculus	nfvPPA>svPPA: none	/	nfvPPA>sbvFTD:<br>R: inf. longitudinal fasciculum, external capsule
**sbvFTD**	sbvFTD>controls:none	sbvFTD>bvFTD:<br>B: ant. and sup. corona radiata, internal capsule, body and genu of corpus callosum, sup.	sbvFTD>svPPA: none	sbvFTD>nfvPPA:<br>L: ant. and sup. corona radiata, internal and external capsule, sup. longitudinal fasciculus	/

		longitudinal fasciculus (L>R)			

Abbreviations: ant.= anterior; B= bilateral; inf.= inferior; L= left; post.= posterior; R= right; sup.= superior.

Supplementary Table 5. Results of TBSS analysis on ODI maps.

**ODI maps**	**Controls**	**bvFTD**	**svPPA**	**nfvPPA**	**sbvFTD**
**Controls**	/	Controls>bvFTD:<br>B: corpus callosum, corona radiata, internal capsule, sup. longitudinal fasciculus	Controls>svPPA:<br>B: internal capsule, corpus callosum, sup. longitudinal fasciculus<br>L: inf. longitudinal fasciculus, cingulum	Controls>nfvPPA:<br>B: corpus callosum, corona radiata, internal capsule, cerebral peduncles, corticospinal tracts<br>L: sup. longitudinal fasciculus	Controls>sbvFTD:<br>R: corona radiata, sup. longitudinal fasciculus
**bvFTD**	bvFTD>controls:<br>B: external capsule, fornix, stria terminalis, inf. longitudinal fasciculus, post. thalamic radiations	/	bvFTD>svPPA:<br>L: inf. longitudinal fasciculus	bvFTD>nfvPPA: none	bvFTD>sbvFTD: none
**svPPA**	svPPA>controls:<br>L: stria terminalis	svPPA>bvFTD: none	/	svPPA>nfvPPA: none	svPPA>sbvFTD: none
**nfvPPA**	nfvPPA>controls: none	nfvPPA>bvFTD: none	nfvPPA>svPPA:<br>L: small cluster of fibres in the inferolateral temporal lobe	/	nfvPPA>sbvFTD: none
**sbvFTD**	sbvFTD>control: none	sbvFTD>bvFTD: none	sbvFTD>svPPA:<br>B: splenium of corpus callosum<br>L: sup. corona radiata	sbvFTD>nfvPPA:<br>L: ant. and sup. corona radiata	/

Abbreviations: ant.= anterior; B= bilateral; inf.= inferior; L= left; post.= posterior; R= right; sup.= superior.

Supplementary Table 6. Mean values of FA, ICVF and ODI for each group for each region of the atlas.

	**Controls**	**bvFTD**	**svPPA**	**nfvPPA**	**sbvFTD**	p value
**GM Cingulate Gyrus L, ICVF**	0.41±0.02 [0.37 - 0.45]	0.40±0.04 [0.34 - 0.57]	0.40±0.03 [0.35 - 0.44]	0.39±0.02 [0.35 - 0.42]	0.41±0.03 [0.37 - 0.45]	0.09
**GM Cingulate Gyrus R, ICVF**	0.41±0.02 [0.37 - 0.45]	0.39±0.03 [0.34 - 0.50]	0.40±0.03 [0.34 - 0.45]	0.39±0.01 [0.37 - 0.42]	0.40±0.03 [0.36 - 0.44]	0.002
**GM Frontal Lobe L, ICVF**	0.53±0.02 [0.47 - 0.57]	0.49±0.05 [0.42 - 0.66]	0.51±0.04 [0.42 - 0.56]	0.48±0.03 [0.45 - 0.51]	0.51±0.03 [0.47 - 0.54]	0.01
**GM Frontal Lobe R, ICVF**	0.52±0.02 [0.48 - 0.59]	0.48±0.05 [0.40 - 0.64]	0.51±0.04 [0.43 - 0.56]	0.50±0.02 [0.45 - 0.53]	0.50±0.03 [0.45 - 0.54]	0.01
**GM Insula L, ICVF**	0.43±0.02 [0.38 - 0.47]	0.40±0.03 [0.34 - 0.45]	0.40±0.04 [0.33 - 0.49]	0.39±0.02 [0.36 - 0.42]	0.42±0.01 [0.40 - 0.43]	<0.001
**GM Insula R, ICVF**	0.43±0.02 [0.39 - 0.47]	0.39±0.03 [0.31 - 0.45]	0.41±0.04 [0.34 - 0.46]	0.40±0.03 [0.34 - 0.45]	0.40±0.04 [0.30 - 0.47]	<0.001
**GM Occipital Lobe L, ICVF**	0.46±0.02 [0.42–0.51]	0.46±0.04 [0.40-0.59]	0.45±0.02 [0.41-0.48]	0.45±0.03 [0.41-0.49]	0.45±0.02 [0.43–0.47]	0.04
**GM Occipital Lobe R, ICVF**	0.45±0.02 [0.41-0.48]	0.45±0.03 [0.39–0.58]	0.44±0.02 [0.40-0.48]	0.45±0.01 [0.43–0.46]	0.43±0.03 [0.41-0.47]	0.17
**GM Parietal Lobe L, ICVF**	0.48±0.03 [0.42-0.54]	0.47±0.04 [0.40-0.59]	0.47±0.03 [0.43–0.53]	0.45±0.04 [0.41-0.54]	0.48±0.03 [0.43-0.50]	0.33
**GM Parietal Lobe R, ICVF**	0.48±0.03 [0.43–0.54]	0.46±0.04 [0.39–0.62]	0.47±0.02 [0.43-0.50]	0.45±0.03 [0.41-0.51]	0.46±0.03 [0.42-0.50]	0.40
**GM Temporal Lobe L, ICVF**	0.45±0.02 [0.41-0.48]	0.44±0.03 [0.39–0.52]	0.41±0.03 [0.36-0.45]	0.43±0.02 [0.40-0.45]	0.43±0.01 "[0.42–0.44]"	<0.001
**GM Temporal Lobe R, ICVF**	0.44±0.02 [0.40-0.47]	0.42±0.05 [0.38–0.66]	0.42±0.03 [0.37-0.45]	0.43±0.01 [0.40-0.45]	0.41±0.02 [0.37-0.45]	0.09
**GM Cingulate Gyrus L, ODI**	0.52±0.02 [0.48-0.56]	0.49±0.05 [0.27–0.53]	0.49±0.03 [0.40-0.52]	0.49±0.02 [0.45-0.52]	0.50±0.02 [0.46-0.53]	<0.001
**GM Cingulate Gyrus R, ODI**	0.50±0.02 [0.45-0.53]	0.46±0.04 [0.25–0.51]	0.48±0.02 [0.43-0.51]	0.48±0.01 [0.47-0.52]	0.47±0.03 [0.40-0.51]	<0.001
**GM Frontal Lobe L, ODI**	0.60±0.02 [0.57-0.64]	0.55±0.03 [0.47-0.60]	0.57±0.02 [0.51-0.60]	0.55±0.04 [0.47-0.61]	0.59±0.02 [0.56-0.63]	<0.001
**GM Frontal Lobe R, ODI**	0.60±0.02 [0.58–0.63]	0.55±0.03 [0.47-0.61]	0.58±0.02 [0.52–0.61]	0.57±0.02 [0.54-0.61]	0.58±0.02 [0.53–0.61]	<0.001
**GM Insula L, ODI**	0.42±0.03 [0.36-0.49]	0.41±0.04 [0.34-0.48]	0.39±0.03 [0.34–0.43]	0.43±0.03 [0.38-0.49]	0.41±0.04 [0.34–0.46]	<0.001
**GM Insula R, ODI**	0.46±0.03 [0.40-0.52]	0.43±0.04 [0.33-0.50]	0.45±0.03 [0.41-0.50]	0.46±0.03 [0.40-0.50]	0.42±0.04 [0.37-0.48]	<0.001
**GM Occipital Lobe L, ODI**	0.61±0.02 [0.57–0.66]	0.59±0.02 [0.53–0.63]	0.59±0.02 [0.55-0.62]	0.60±0.02 [0.55-0.63]	0.60±0.02 [0.57-0.64]	<0.001
**GM Occipital Lobe R, ODI**	0.60±0.02 [0.55-0.63]	0.58±0.02 [0.53–0.62]	0.59±0.01 [0.56–0.62]	0.59±0.02 [0.57-0.62]	0.58±0.03 [0.54–0.62]	0.02
**GM Parietal Lobe L, ODI**	0.61±0.02 [0.57-0.65]	0.58±0.03 [0.51–0.62]]	0.57±0.03 [0.51–0.61]	0.56±0.04 [0.45–0.63]]	0.59±0.02 [0.56-0.63]	<0.001
**GM Parietal Lobe R, ODI**	0.60±0.02 [0.55-0.64]	0.56±0.03 [0.49-0.63]	0.57±0.03 [0.51-0.62]	0.57±0.03 [0.53–0.64]	0.57±0.02 [0.55–0.61]	<0.001
**GM Temporal Lobe L, ODI**	0.55±0.01 [0.51–0.58]	0.53±0.03 [0.45-0.56]	0.49±0.02 [0.45-0.52]	0.53±0.02 [0.48-0.56]	0.53±0.02 [0.50-0.55]	<0.001
**GM Temporal Lobe R, ODI**	0.54±0.01 [0.52–0.57]]	0.52±0.03 [0.42–0.56]	0.53±0.02 [0.47-0.55]	0.54±0.01 [0.52–0.56]	0.49±0.02 [0.46-0.53]	<0.001
**WM Cingulate Gyrus L, ICVF**	0.55±0.03 [0.50–0.62]	0.52±0.06 [0.43-0.69]	0.52±0.05 [0.43-0.63]	0.51±0.05 [0.40-0.57]	0.54±0.03 [0.50-0.58]	0.01
**WM Cingulate Gyrus R, ICVF**	0.52±0.03 [0.47-0.59]	0.48±0.05 [0.40-0.68]	0.51±0.04 [0.43-0.60]	0.50±0.03 [0.44-0.54]	0.50±0.03 [0.43-0.53]	0.01
**WM Corpus Callosum ICVF**	0.64±0.04 [0.54-0.73]	0.61±0.07 [0.51-0.78]	0.64±0.05 [0.56-0.75]	0.60±0.05 [0.50-0.67]	0.63±0.03 [0.59–0.68]	0.07
**WM Frontal Lobe L, ICVF**	0.55±0.03 [0.48-0.61]	0.50±0.05 [0.39–0.67]	0.53±0.06 [0.40–0.62]	0.49±0.05 [0.41-0.57]	0.56±0.02 [0.52–0.57]	<0.001
**WM Frontal Lobe R, ICVF**	0.54±0.03 [0.47-0.60]	0.48±0.05 [0.39–0.63]	0.53±0.05 [0.43-0.62]	0.51±0.04 [0.45-0.56]	0.53±0.02 [0.48-0.56]	<0.001
**WM Insula L, ICVF**	0.44±0.02 [0.38–0.48]	0.40±0.04 [0.33-0.54]	0.40±0.05 [0.28-0.47]	0.40±0.02 [0.37-0.44]	0.43±0.03 [0.37–0.47]	<0.001

**WM Insula R, ICVF**	0.46±0.02 [0.40 - 0.51]	0.40±0.04 [0.33 - 0.55]	0.44±0.05 [0.33 - 0.51]	0.43±0.02 [0.39 - 0.46]	0.41±0.03 [0.33 - 0.44]	<0.001
**WM Occipital Lobe L, ICVF**	0.54±0.03 [0.48 - 0.62]	0.54±0.04 [0.48 - 0.63]	0.53±0.04 [0.43 - 0.60]	0.54±0.03 [0.49 - 0.58]	0.54±0.02 [0.50 - 0.57]	0.85
**WM Occipital Lobe R, ICVF**	0.54±0.03 [0.48 - 0.61]	0.54±0.04 [0.46 - 0.65]	0.53±0.04 [0.43 - 0.60]	0.54±0.03 [0.48 - 0.58]	0.53±0.02 [0.50 - 0.57]	0.68
**WM Parietal Lobe L, ICVF**	0.59±0.03 [0.53 - 0.65]	0.56±0.04 [0.49 - 0.66]	0.57±0.05 [0.45 - 0.65]	0.55±0.04 [0.46 - 0.6]	0.58±0.02 [0.56 - 0.62]	0.04
**WM Parietal Lobe R, ICVF**	0.58±0.03 [0.51 - 0.64]	0.55±0.04 [0.48 - 0.67]	0.57±0.05 [0.47 - 0.66]	0.56±0.04 [0.49 - 0.61]	0.56±0.02 [0.54 - 0.59]	0.09
**WM Temporal Lobe L, ICVF**	0.50±0.03 [0.44 - 0.57]	0.47±0.04 [0.39 - 0.56]	0.43±0.04 [0.35 - 0.51]	0.47±0.03 [0.40 - 0.52]	0.47±0.02 [0.45 - 0.5]	<0.001
**WM Temporal Lobe R, ICVF**	0.49±0.03 [0.43 - 0.53]	0.46±0.04 [0.35 - 0.55]	0.46±0.04 [0.37 - 0.53]	0.47±0.03 [0.42 - 0.53]	0.41±0.02 [0.39 - 0.44]	<0.001
**WM Cingulate Gyrus L, ODI**	0.19±0.01 [0.17 - 0.21]	0.20±0.01 [0.16 - 0.23]	0.19±0.01 [0.17 - 0.23]	0.19±0.01 [0.18 - 0.20]	0.19±0.01 [0.18 - 0.22]	0.22
**WM Cingulate Gyrus R, ODI**	0.22±0.01 [0.19 - 0.25]	0.22±0.01 [0.19 - 0.25]	0.22±0.02 [0.20 - 0.27]	0.22±0.01 [0.21 - 0.23]	0.22±0.01 [0.21 - 0.24]	0.19
**WM Corpus Callosum ODI**	0.07±0.01 [0.05 - 0.08]	0.06±0.01 [0.04 - 0.08]	0.06±0.01 [0.05 - 0.09]	0.06±0.01 [0.04 - 0.07]	0.06±0.01 [0.05 - 0.08]	0.003
**WM Frontal Lobe L, ODI**	0.25±0.01 [0.24 - 0.26]	0.24±0.01 [0.23 - 0.26]	0.25±0.01 [0.23 - 0.28]	0.24±0.01 [0.23 - 0.26]	0.25±0.01 [0.24 - 0.26]	0.23
**WM Frontal Lobe R, ODI**	0.25±0.01 [0.24 - 0.27]	0.24±0.01 [0.23 - 0.26]	0.25±0.01 [0.23 - 0.30]	0.25±0.01 [0.23 - 0.25]	0.25±0.01 [0.24 - 0.26]	0.31
**WM Insula L, ODI**	0.27±0.01 [0.25 - 0.32]	0.29±0.02 [0.25 - 0.33]	0.29±0.02 [0.23 - 0.34]	0.30±0.02 [0.25 - 0.33]	0.28±0.02 [0.25 - 0.29]	<0.001
**WM Insula R, ODI**	0.27±0.02 [0.24 - 0.31]	0.28±0.02 [0.24 - 0.33]	0.28±0.02 [0.24 - 0.33]	0.28±0.01 [0.25 - 0.29]	0.28±0.02 [0.26 - 0.30]	0.13
**WM Occipital Lobe L, ODI**	0.26±0.01 [0.24 - 0.27]	0.28±0.01 [0.25 - 0.30]	0.27±0.02 [0.24 - 0.32]	0.27±0.01 [0.26 - 0.29]	0.27±0.01 [0.26 - 0.29]	<0.001
**WM Occipital Lobe R, ODI**	0.27±0.01 [0.25 - 0.29]	0.29±0.01 [0.26 - 0.32]	0.28±0.02 [0.25 - 0.34]	0.28±0.01 [0.26 - 0.3]	0.28±0.01 [0.27 - 0.3]	<0.001
**WM Parietal Lobe L, ODI**	0.23±0.01 [0.21 - 0.24]	0.23±0.01 [0.21 - 0.25]	0.23±0.01 [0.21 - 0.27]	0.22±0.01 [0.21 - 0.23]	0.23±0.01 [0.22 - 0.24]	0.06
**WM Parietal Lobe R, ODI**	0.23±0.01 [0.22 - 0.25]	0.23±0.01 [0.21 - 0.25]	0.23±0.02 [0.22 - 0.28]	0.23±0.01 [0.22 - 0.24]	0.23±0.01 [0.22 - 0.24]	0.83
**WM Temporal Lobe L, ODI**	0.25±0.01 [0.23 - 0.27]	0.25±0.01 [0.23 - 0.27]	0.24±0.01 [0.22 - 0.26]	0.25±0.01 [0.24 - 0.27]	0.25±0.01 [0.24 - 0.26]	<0.001
**WM Temporal Lobe R, ODI**	0.25±0.01 [0.23 - 0.27]	0.26±0.01 [0.23 - 0.29]	0.25±0.01 [0.23 - 0.30]	0.26±0.01 [0.24 - 0.27]	0.25±0.01 [0.24 - 0.28]	0.06
**WM Cingulate Gyrus L, FA**	0.53±0.02 [0.49 - 0.58]	0.50±0.04 [0.40 - 0.57]	0.51±0.04 [0.44 - 0.56]	0.50±0.02 [0.45 - 0.53]	0.52±0.03 [0.46 - 0.57]	0.000
**WM Cingulate Gyrus R, FA**	0.48±0.02 [0.42 - 0.53]	0.45±0.03 [0.39 - 0.51]	0.47±0.04 [0.40 - 0.52]	0.47±0.02 [0.44 - 0.49]	0.46±0.03 [0.40 - 0.50]	<0.001
**WM Corpus Callosum FA**	0.75±0.03 [0.68 - 0.80]	0.72±0.04 [0.61 - 0.79]	0.74±0.04 [0.67 - 0.8]	0.72±0.03 [0.66 - 0.78]	0.74±0.04 [0.67 - 0.81]	0.001
**WM Frontal Lobe L, FA**	0.45±0.02 [0.42 - 0.49]	0.42±0.03 [0.35 - 0.46]	0.44±0.03 [0.37 - 0.48]	0.42±0.03 [0.37 - 0.46]	0.45±0.02 [0.43 - 0.47]	<0.001
**WM Frontal Lobe R, FA**	0.45±0.02 [0.41 - 0.48]	0.41±0.03 [0.36 - 0.45]	0.44±0.03 [0.37 - 0.49]	0.43±0.02 [0.39 - 0.46]	0.44±0.02 [0.41 - 0.47]	<0.001
**WM Insula L, FA**	0.36±0.02 [0.32 - 0.40]	0.33±0.03 [0.28 - 0.37]	0.33±0.03 [0.28 - 0.38]	0.33±0.02 [0.29 - 0.37]	0.35±0.02 [0.31 - 0.38]	<0.001
**WM Insula R, FA**	0.38±0.02 [0.34 - 0.41]	0.33±0.03 [0.28 - 0.41]	0.36±0.03 [0.31 - 0.41]	0.36±0.01 [0.34 - 0.38]	0.34±0.03 [0.30 - 0.37]	<0.001
**WM Occipital Lobe L, FA**	0.44±0.02 [0.40 - 0.48]	0.42±0.02 [0.37 - 0.45]	0.43±0.04 [0.34 - 0.48]	0.42±0.02 [0.40 - 0.45]	0.43±0.01 [0.41 - 0.45]	0.001
**WM Occipital Lobe R, FA**	0.43±0.02 [0.38 - 0.46]	0.40±0.02 [0.32 - 0.44]	0.41±0.04 [0.33 - 0.47]	0.41±0.02 [0.38 - 0.44]	0.41±0.01 [0.39 - 0.43]	0.001
**WM Parietal Lobe L, FA**	0.49±0.02 [0.46 - 0.53]	0.47±0.02 [0.42 - 0.51]	0.48±0.03 [0.40 - 0.52]	0.48±0.02 [0.43 - 0.50]	0.48±0.01 [0.47 - 0.51]	0.02
**WM Parietal Lobe R, FA**	0.48±0.02 [0.45 - 0.52]	0.46±0.02 [0.41 - 0.51]	0.47±0.03 [0.40 - 0.52]	0.47±0.02 [0.44 - 0.50]	0.47±0.01 [0.45 - 0.5]	0.01

**WM Temporal Lobe L, FA**	0.43±0.02 [0.40 - 0.47]	0.40±0.02 [0.36 - 0.44]	0.39±0.03 [0.32 - 0.43]	0.41±0.02 [0.37 - 0.44]	0.40±0.01 [0.38 - 0.42]	<0.001
**WM Temporal Lobe R, FA**	0.43±0.01 [0.39 - 0.45]	0.39±0.02 [0.33 - 0.43]	0.40±0.03 [0.33 - 0.45]	0.41±0.02 [0.38 - 0.44]	0.37±0.02 [0.33 - 0.40]	<0.001
**WM Cingulate Gyrus L, MD**	0.00052 ± 0.00002 [0.00049 - 0.00055]	0.00055 ± 0.00003 [0.00050 - 0.00062]	0.00055 ± 0.00003 [0.00051 - 0.00062]	0.00054 ± 0.00003 [0.00049 - 0.00061]	0.00053 ± 0.00002 [0.00051 - 0.00057]	<0.001
**WM Cingulate Gyrus R, MD**	0.00054 ± 0.00002 [0.00050 - 0.00057]	0.00057 ± 0.00002 [0.00052 - 0.00062]	0.00056 ± 0.00002 [0.00053 - 0.00060]	0.00055 ± 0.00003 [0.00051 - 0.00061]	0.00056 ± 0.00003 [0.00053 - 0.00064]	<0.001
**WM Corpus Callosum MD**	0.00051 ± 0.00002 [0.00047 - 0.00055]	0.00056 ± 0.00004 [0.00049 - 0.00066]	0.00055 ± 0.00004 [0.00050 - 0.00061]	0.00054 ± 0.00004 [0.00047 - 0.00063]	0.00053 ± 0.00003 [0.00050 - 0.00058]	<0.001
**WM Frontal Lobe L, MD**	0.00050 ± 0.00002 [0.00047 - 0.00055]	0.00055 ± 0.00004 [0.00049 - 0.00065]	0.00055 ± 0.00004 [0.00050 - 0.00061]	0.00053 ± 0.00004 [0.00048 - 0.00062]	0.00051 ± 0.00001 [0.00049 - 0.00052]	<0.001
**WM Frontal Lobe R, MD**	0.00051 ± 0.00002 [0.00048 - 0.00055]	0.00056 ± 0.00003 [0.00051 - 0.00062]	0.00053 ± 0.00003 [0.00050 - 0.00058]	0.00052 ± 0.00003 [0.00047 - 0.00059]	0.00052 ± 0.00002 [0.00050 - 0.00057]	<0.001
**WM Insula L, MD**	0.00060 ± 0.00002 [0.00057 - 0.00064]	0.00066 ± 0.00004 [0.00059 - 0.00076]	0.00064 ± 0.00002 [0.00061 - 0.00068]	0.00066 ± 0.00005 [0.00060 - 0.00079]	0.00063 ± 0.00004 [0.00060 - 0.00072]	<0.001
**WM Insula R, MD**	0.00058 ± 0.00002 [0.00054 - 0.00062]	0.00065 ± 0.00004 [0.00057 - 0.00073]	0.00061 ± 0.00002 [0.00058 - 0.00064]	0.00061 ± 0.00004 [0.00055 - 0.00070]	0.00063 ± 0.00005 [0.00058 - 0.00075]	<0.001
**WM Occipital Lobe L, MD**	0.00052 ± 0.00002 [0.00048 - 0.00056]	0.00052 ± 0.00002 [0.00049 - 0.00056]	0.00052 ± 0.00002 [0.00050 - 0.00056]	0.00053 ± 0.00003 [0.00049 - 0.00059]	0.00052 ± 0.00001 [0.00051 - 0.00055]	0.63
**WM Occipital Lobe R, MD**	0.00052 ± 0.00002 [0.00048 - 0.00056]	0.00052 ± 0.00002 [0.00049 - 0.00059]	0.00052 ± 0.00002 [0.00049 - 0.00056]	0.00053 ± 0.00003 [0.00049 - 0.00060]	0.00052 ± 0.00001 [0.00051 - 0.00054]	0.57
**WM Parietal Lobe L, MD**	0.00050 ± 0.00002 [0.00046 - 0.00053]	0.00052 ± 0.00002 [0.00048 - 0.00056]	0.00053 ± 0.00003 [0.00049 - 0.00058]	0.00052 ± 0.00003 [0.00047 - 0.00060]	0.00050 ± 0.00001 [0.00048 - 0.00052]	0.001
**WM Parietal Lobe R, MD**	0.00050 ± 0.00002 [0.00046 - 0.00053]	0.00052 ± 0.00002 [0.00048 - 0.00057]	0.00052 ± 0.00002 [0.00049 - 0.00056]	0.00051 ± 0.00003 [0.00046 - 0.00058]	0.00051 ± 0.00001 [0.00050 - 0.00053]	0.002
**WM Temporal Lobe L, MD**	0.00055 ± 0.00002 [0.00052 - 0.00059]	0.00058 ± 0.00003 [0.00053 - 0.00066]	0.00058 ± 0.00002 [0.00055 - 0.00063]	0.00063 ± 0.00004 [0.00056 - 0.00071]	0.00058 ± 0.00002 [0.00056 - 0.00061]	<0.001
**WM Temporal Lobe R, MD**	0.00055 ± 0.00002 [0.00052 - 0.00060]	0.00059 ± 0.00004 [0.00053 - 0.00074]	0.00057 ± 0.00002 [0.00053 - 0.00060]	0.00058 ± 0.00004 [0.00053 - 0.00067]	0.00064 ± 0.00003 [0.00061 - 0.00072]	<0.001

Values are reported as means ± standard deviations [min – max]; p values refer to the significance of the effect of the group category (F-test) in a regression model (ANCOVA) that considered age, sex and education as covariates. Abbreviations: bvFTD = behavioral variant frontotemporal dementia; FA = fractional anisotropy; GM = gray matter; ICVF = intra-cellular fractional volume; L= left; nfvPPA = nonfluent/agrammatic variant Primary Progressive Aphasia; ODI = orientation-dispersion index; R= right; sbvFTD = semantic behavioral variant Frontotemporal Dementia; svPPA = semantic variant Primary Progressive Aphasia, WM = white matter.

Supplementary Table 7. Results of GBSS analysis on ICVF maps.

ICVF maps	Controls	bvFTD	svPPA	nfvPPA	sbvFTD
**Controls**	/	Controls>bvFTD:<br>B: whole-frontal lobe, insula, anterior temporal lobe, including the amygdala, sparing the hippocampal and parahippocampal region. bilateral putamen and caudate nucleus<br>R: superior and middle temporal pole	Controls>svPPA:<br>L: whole temporal lobe cortex, anterior cingulum and left caudatum	Controls>nfvPPA:<br>L: frontal, temporal and anterior parietal lobes, with a relative sparing of medial orbitofrontal cortex	Controls>sbvFTD:<br>R: insula, olfactory cortex, hippocampus, middle temporal and inferior occipital cortex, including fusiform gyrus
**bvFTD**	bvFTD > controls: none	/	bvFTD>svPPA:<br>none	bvFTD>nfvPPA:<br>L: middle occipital lobe	bvFTD>sbvFTD:none
**svPPA**	svPPA>Controls:none	svPPA>bvFTD:<br>R: frontal superior and inferior orbital cortex, insula	/	svPPA>nfvPPA:<br>L: superior frontal lobe	svPPA>sbvFTD: none
**nfvPPA**	nfvPPA>Controls: none	nfvPPA>bvFTD:<br>none	nfvPPA>svPPA:<br>none	/	nfvPPA>sbvFTD: none
**sbvFTD**	sbvFTD>Controls:none	sbvFTD>bvFTD:<br>none	sbvFTD>svPPA:<br>none	sbvFTD>nfvPPA: none	/

Abbreviations: B=bilateral; L= left; R= right.

Supplementary Table 8. Results of GBSS analysis on ODI maps.

**ODI maps**	**Controls**	**bvFTD**	**svPPA**	**nfvPPA**	**sbvFTD**
**Controls**	/	Controls>bvFTD:<br>B: widespread damage, including frontal, parietal, temporal, occipital lobes, with relative sparing of inferior temporal lobes, bilateral putamen and caudate	Controls>svPPA:<br>L: diffuse frontal temporal, and parietal lobes, with a relative sparing of occipital cortex, but involving precuneus and cingulate cortex.<br>R: orbitofrontal, superior frontal cortex, supramarginal and postcentral gyri, superior parietal lobe and right caudate	Controls>nfvPPA:<br>L: diffuse frontal temporal and parietal lobes, middle occipital lobe, cingulum, precuneus<br>R: superior frontal and parietal lobes, precuneus	Controls>sbvFTD:<br>R: insula, medial anterior and inferior temporal lobe, middle frontal and parietal, middle occipital, middle cingulate cortex
**bvFTD**	bvFTD>controls: none	/	bvFTD>svPPA:<br>L: amygdala and hippocampus	bvFTD>nfvPPA: none	bvFTD>sbvFTD: none
**svPPA**	svPPA>controls: none	svPPA>bvFTD:<br>R: diffuse frontal lobe, insula	/	svPPA>nfvPPA: none	svPPA>sbvFTD: none
**nfvPPA**	nfvPPA>controls:none	nfvPPA>bvFTD:<br>R: superior frontal lobe, insula, caudate, putamen	nfvPPA>svPPA:<br>L: hippocampal and parahippocampal regions, amygdala, anterior temporal pole	/	nfvPPA>sbvFTD:<br>R: small clusters on amygdala, and parahippocampal regions
**sbvFTD**	sbvFTD>controls:none	sbvFTD>bvFTD:<br>B: superior frontal cortex<br>L: postcentral gyrus	sbvFTD>svPPA: none	sbvFTD>nfvPPA:<br>L: superior frontal, precentral, postcentral gyri, inferior parietal	/

Abbreviations: B=bilateral; L= left; R= right.

Supplementary Table 9. Correlation analysis results across patient groups.

Clinical scale	Neuroimaging feature	r	P value	Adjusted P value
bvFTD				
MMSE	WM Temporal Lobe L MD	-0.64	0.004	0.054
MMSE	WM Parietal Lobe L MD	-0.62	0.01	0.08
MMSE	WM Temporal Lobe L ICVF	0.60	<0.01	0.11
MMSE	WM Parietal Lobe L ICVF	0.56	0.02	0.22
MMSE	GM Temporal Lobe L ODI	0.56	0.02	0.21
MMSE	GM Parietal Lobe L ODI	0.54	0.02	0.24
MMSE	GM Occipital Lobe L ODI	0.52	0.03	0.33
MMSE	GM Parietal Lobe L ICVF	0.50	0.04	0.43
svPPA				
CDR-sb	GM Insula R ICVF	-0.77	<0.01	0.04
CDR-sb	GM Temporal Lobe R ICVF	-0.76	<0.01	0.05
CDR-sb	WM Cingulate Gyrus R MD	0.76	0.01	0.08
CDR-sb	WM Insula R ICVF	-0.72	<0.01	0.11
CDR-sb	WM Insula R MD	0.69	0.02	0.23
CDR-sb	WM Temporal Lobe R FA	-0.68	0.02	0.21
CDR-sb	WM Temporal Lobe R MD	0.67	0.02	0.30
CDR-sb	GM Temporal Lobe L ICVF	-0.67	0.02	0.19
CDR-sb	WM Parietal Lobe L FA	-0.66	0.02	0.25
CDR-sb	GM Frontal Lobe R ICVF	-0.65	0.02	0.25
MMSE	WM Parietal Lobe L ODI	-0.65	0.03	0.39
CDR-sb	GM Cingulate Gyrus R ICVF	-0.62	0.03	0.39
CDR-sb	WM Cingulate Gyrus R FA	-0.60	0.04	0.50
CDR-sb	GM Frontal Lobe L ICVF	-0.60	0.04	0.47
MMSE	WM Insula L MD	0.61	0.048	0.62
CDR-sb	WM Frontal Lobe L FA	-0.59	0.04	0.54
CDR-sb	WM Cingulate Gyrus R ICVF	-0.58	0.05	0.60
CDR-sb	WM Occipital Lobe R FA	-0.58	0.05	0.62
nfvPPA				
CDR-sb	GM Temporal Lobe L ODI	-0.90	<0.01	0.02
MMSE	GM Temporal Lobe L ODI	0.90	<0.01	0.06
CDR-sb	WM Occipital Lobe R ODI	0.84	<0.01	0.11
CDR-sb	GM Parietal Lobe L ODI	-0.74	0.03	0.42
CDR-sb	GM Cingulate Gyrus L ODI	-0.74	0.04	0.43
sbvFTD				
CDR-sb	WM Cingulate Gyrus L MD	0.97	0.004	0.06
CDR-sb	WM Cingulate Gyrus R ODI	0.92	0.03	0.33
MMSE	WM Cingulate Gyrus L ODI	-0.91	0.03	0.41
CDR-sb	WM Temporal Lobe L MD	0.91	0.03	0.44
MMSE	WM Corpus Callosum ODI	-0.89	0.04	0.52

CDR-sb	GM Temporal Lobe L ODI	-0.89	0.04	0.51

Abbreviations: bvFTD = behavioral variant frontotemporal dementia; CDR-sb = Clinical Dementia Rating sum of boxes; FA = fractional anisotropy; GM = gray matter; ICVF = intra-cellular fractional volume; L= left; MD= Mean diffusivity; MMSE = Mini-mental-state evaluation; nfvPPA = nonfluent/agrammatic variant Primary Progressive Aphasia; ODI = orientation-dispersion index; R= right; sbvFTD = semantic behavioral variant Frontotemporal Dementia; svPPA = semantic variant Primary Progressive Aphasia, WM = white matter.

Supplementary Table 10. Comparative analysis of machine learning model performance using different feature combinations.

**Feature combination**	**Models metrics**				**Metrics of models following the feature selection**			
	Accuracy	Precision	Recall	F1- score	Accuracy	Precision	Recall	F1-score
FA + MD	75.3%	75.5%	73.4%	74.3%	86.3%	87.7%	84.6%	85.4%
FA +ICVF	69.9%	66.7%	70.1%	67.8%	83.6%	82.7%	84.1%	83.0%
FA + ODI	65.8%	64.2%	62.9%	63.5%	89.0%	89.6%	89.8%	89.5%
FA + PSI	61.6%	59.5%	60.6%	59.9%	86.3%	87.9%	84.7%	85.9%
MD + ICVF	79.5%	77.6%	77.5%	77.5%	91.8%	92.6%	89.1%	90.5%
MD + ODI	69.9%	68.2%	67.5%	67.8%	87.7%	87.2%	85.6%	86.2%
MD + PSI	83.6%	81.5%	81.8%	81.4%	91.8%	90.3%	91.4%	90.8%
ICVF+ODI	64.4%	61.3%	64.1%	62.3%	89.0%	89.0%	88.7%	88.6%
ICVF+PSI	80.8%	75.4%	76.8%	75.5%	93.2%	91.3%	90.9%	90.9%
ODI+PSI	64.4%	61.5%	63.5%	62.4%	83.6%	79.4%	79.5%	79.1%
FA + MD + ICVF	76.7%	74.5%	75.4%	74.6%	86.3%	84.1%	83.6%	83.8%
FA + MD + ODI	69.9%	67.6%	67.5%	67.5%	87.7%	91.5%	83.8%	86.9%
FA + MD + PSI	83.6%	81.6%	81.2%	81.3%	95.9%	96.1%	95.7%	95.8%
FA + ICVF + ODI	68.5%	67.9%	71.0%	69.2%	94.5%	96.5%	93.2%	94.7%
FA + ICVF + PSI	79.5%	77.1%	78.4%	77.2%	94.5%	93.5%	92.3%	92.8%
FA + ODI + PSI	71.2%	67.9%	66.9%	67.1%	93.2%	92.2%	91.5%	91.8%
MD + ICVF + ODI	68.5%	66.0%	69.6%	67.4%	93.2%	94.0%	93.0%	93.4%

MD + ICVF + PSI	83.6%	80.8%	83.6%	82.0%	97.3%	98.6%	95.8%	97.1%
MD + ODI + PSI	78.1%	71.9%	72.2%	71.8%	90.4%	88.4%	90.0%	89.1%
ICVF+ODI+PSI	74.0%	69.1%	69.6%	69.2%	97.3%	98.6%	95.3%	96.8%

Abbreviations: FA = fractional anisotropy; MD = mean diffusivity; ICVF = intra-cellular fractional volume, ODI = orientation-dispersion index; PSI = neuropsychological data.

Supplementary Table 11. Features included in the best machine learning model.

White matter derived features (mean value of ROIs obtained from white matter skeleton)
Right temporal lobe, FA
Left frontal lobe, ODI
Right frontal lobe, ICVF
Left insula, ODI
Right insula, ICVF
Right cingulate gyrus, ODI
Left temporal lobe, ODI
Right temporal lobe, ICVF
Left parietal lobe, ICVF
Right parietal lobe, ODI
Corpus callosum, ODI
Gray Matter derived features (mean value of ROIs obtained from gray matter skeleton)
Right frontal lobe, ODI
Right insula, ICVF
Left cingulate gyrus, ICVF
Left temporal lobe, ICVF
Left temporal lobe, ODI
Right temporal lobe, ODI
Right parietal lobe, ICVF
Left parietal lobe, ODI
Right parietal lobe, ODI
Left occipital lobe, ICVF
Right occipital lobe, ICVF
Right occipital lobe, ODI
Neuropsychological data
Attentive matrices
FAB
FBI-A
FBI-B
Digit span, backwards
Semantic fluency
SET-Emotion Attribution
Token test

Abbreviations: FAB = frontal assessment battery; FBI = Frontal behavioural inventory; ICVF = intra-cellular fractional volume; ODI = orientation-dispersion index; SET = Story-based empathy task.

Supplementary Table 12. Comparative analysis of multiple machine learning algorithms accuracy.

	**Accuracy before feature selection**				**Accuracy after feature selection and hyperparameter tuning**			
**Features combination**	Random Forest	XGBoost	Naive Bayes	Logistic regression	Random Forest	XGBoost	Naive Bayes	Logistic regression
FA	53.4%	45.2%	46.6%	53.4%	50.7%	50.7%	58.9%	74.0%
ICVF	61.6%	69.9%	50.7%	71.2%	64.4%	69.9%	71.2%	80.8%
ODI	61.6%	63.0%	54.8%	63.0%	67.1%	69.9%	63.0%	82.2%
PSI	61.6%	60.3%	47.9%	50.7%	65.8%	64.4%	64.4%	61.6%
FA+ICVF	63.0%	64.4%	47.9%	65.8%	63.0%	76.7%	61.6%	87.7%
FA+ODI	56.2%	60.3%	53.4%	63.0%	60.3%	65.8%	71.2%	83.6%
FA + PSI	63.0%	71.2%	50.7%	57.5%	71.2%	79.5%	61.6%	79.5%
ICVF+ODI	61.6%	72.6%	57.5%	69.9%	68.5%	67.1%	72.6%	82.2%
ICVF+PSI	68.5%	82.2%	58.9%	79.5%	76.7%	83.6%	75.3%	91.8%
ODI+PSI	69.9%	79.5%	56.2%	71.2%	69.9%	76.7%	67.1%	83.6%
FA+ICVF+ODI	52.1%	75.3%	57.5%	65.8%	58.9%	74.0%	71.2%	86.3%
FA+ICVF+PSI	68.5%	76.7%	56.2%	74.0%	76.7%	79.5%	83.6%	86.3%
FA+ODI+PSI	64.4%	69.9%	58.9%	74.0%	65.8%	83.6%	74.0%	90.4%
ICVF+ODI+PSI	72.6%	80.8%	67.1%	72.6%	75.3%	82.2%	78.1%	93.2%
FA+ICVF+ODI+PSI	64.4%	79.5%	56.2%	68.5%	74.0%	89.0%	75.3%	89.0%

Abbreviations: FA = fractional anisotropy; ICVF = intra-cellular fractional volume, ODI = orientation-dispersion index; PSI = neuropsychological data.

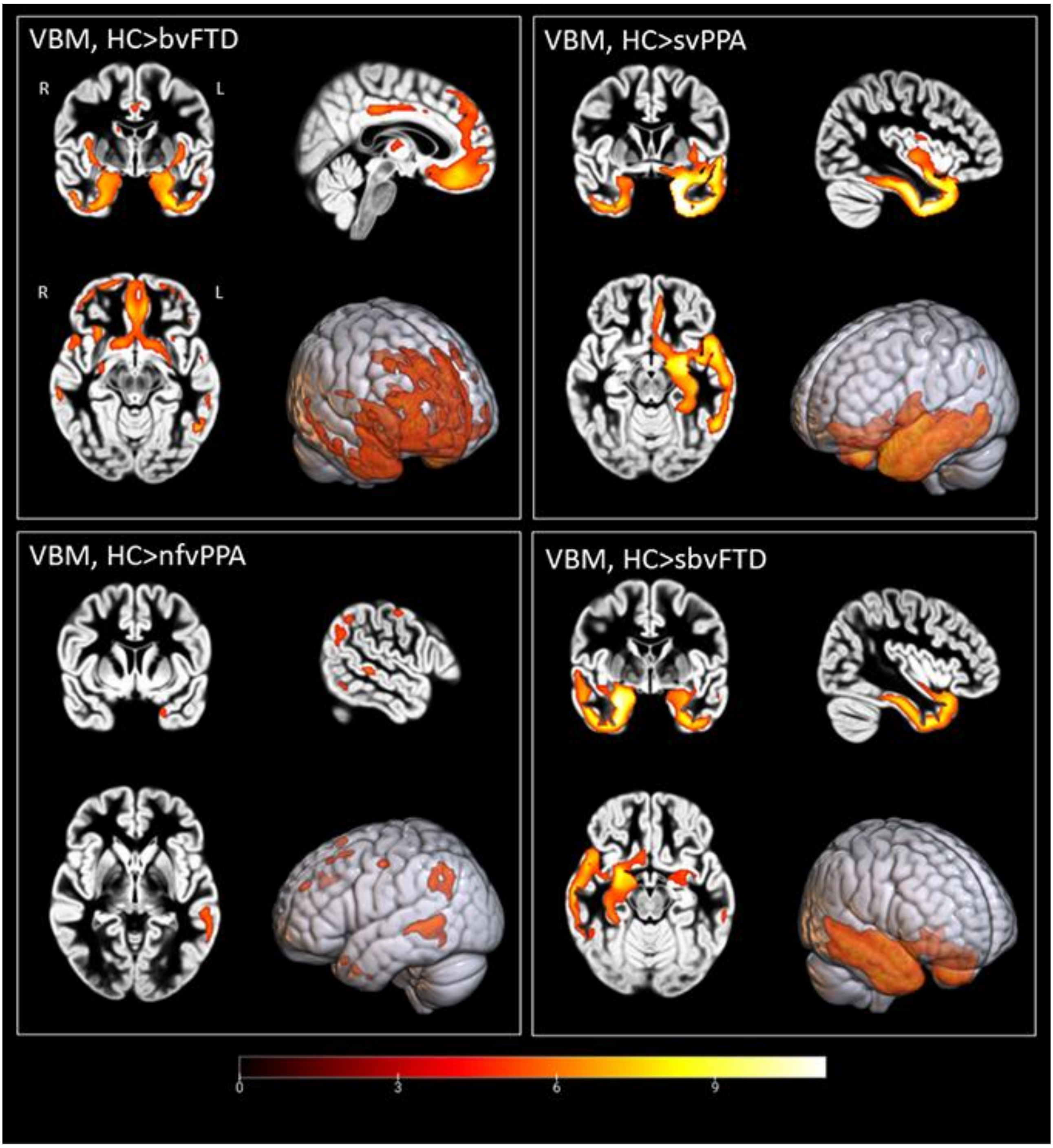

Supplementary Figure 1. Voxel-based morphometry results in bvFTD patients, svPPA patients, nfvPPA patients, sbvFTD patients versus healthy controls. Regions of gray matter atrophy in FTD patients compared to controls are shown in yellow-to-red and overlaid on the coronal, sagittal and axial sections of the Montreal Neurological Institute standard brain in radiological convention (right is left). Results are displayed at $p<0.05$ Family-wise error corrected for multiple comparisons, adjusting for age, sex, education and total intracranial volume. Color bar refers to T values. Abbreviations: L= left; R= right.

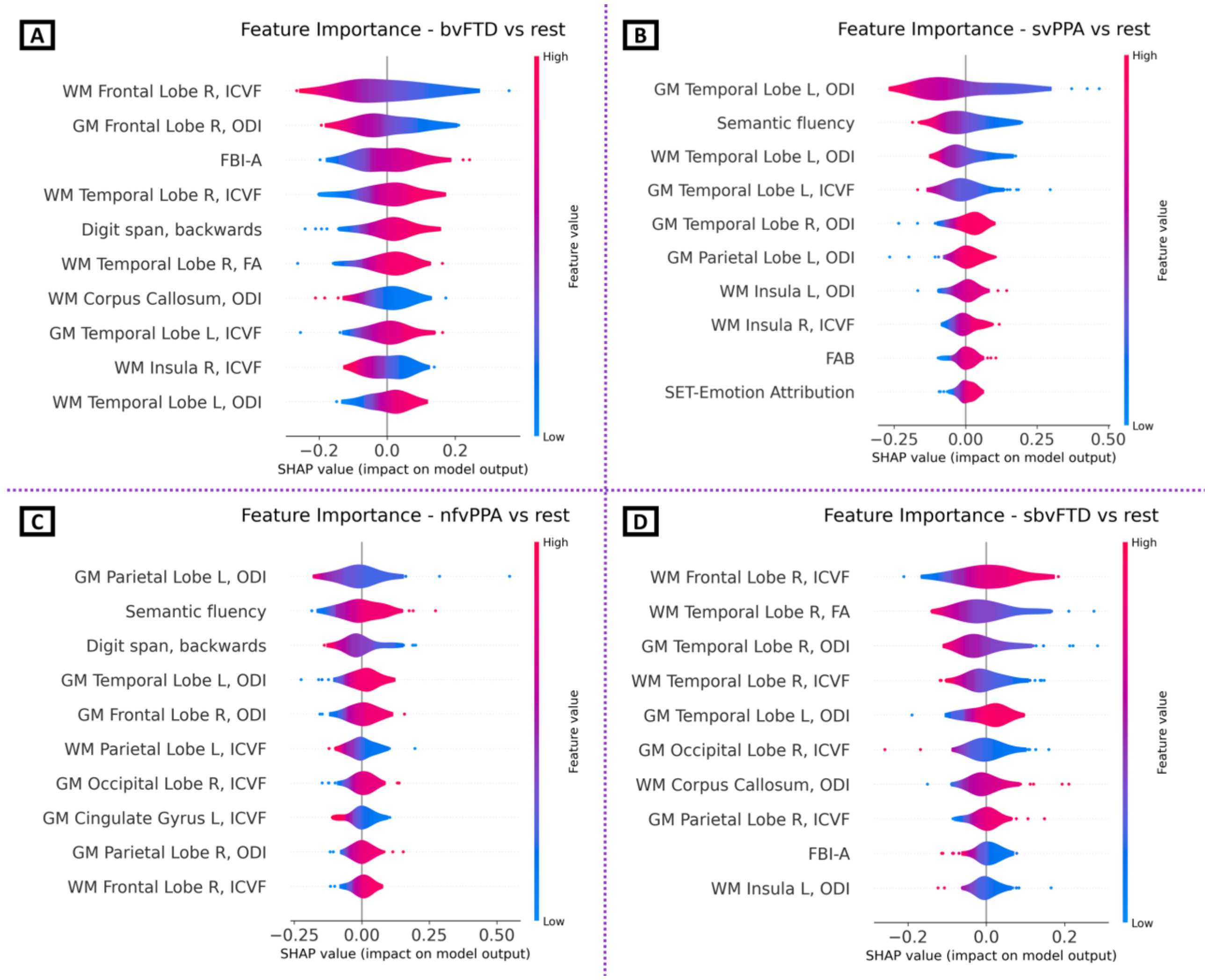

Supplementary Figure 2. Multiclass classification. SHAP summary plot is the representation of the importance of each feature in subject-specific classification highlighting their influence on syndrome classification. The color of the dots is indicating how the sign of the feature is contributing to the prediction (red indicates higher values; blue indicates lower values). SHAP value is the amount the predictor contributes to the model output (Number of subject=73). *Abbreviations: FBI = Frontal behavioural inventory; GM = gray matter; ICVF = intra-cellular fractional volume; L= left; ODI = orientation-dispersion index; R= right; WM = white matter.*
